# CK2 derived from brain microvascular endothelial cells induces astrocyte inflammatory response in *Escherichia coli*-induced meningitis

**DOI:** 10.1371/journal.ppat.1013464

**Published:** 2025-09-10

**Authors:** Dong Huo, Ruicheng Yang, Jiyang Fu, Jiaqi Chen, Chen Tan, Huanchun Chen, Xiangru Wang

**Affiliations:** 1 National Key Laboratory of Agricultural Microbiology, College of Veterinary Medicine, Huazhong Agricultural University, Wuhan, China; 2 Key Laboratory of Preventive Veterinary Medicine in Hubei Province, The Cooperative Innovation Center for Sustainable Pig Production, Huazhong Agricultural University, Wuhan, China; 3 Institute of Microalgae Synthetic Biology and Green Manufacturing, School of Life Sciences, Jianghan University, Wuhan, China; 4 Engineering Research Center of Animal Biopharmaceuticals, The Ministry of Education of the People’s Republic of China (MOE), Wuhan, China; 5 Frontiers Science Center for Animal Breeding and Sustainable Production, Huazhong Agricultural University, Wuhan, China; University of Maryland School of Medicine, UNITED STATES OF AMERICA

## Abstract

Neuroinflammation within the central nervous system (CNS) is recognized as a critical pathological process in meningitic *Escherichia coli* (*E. coli*) infection, leading to severe neurodegenerative disorders and long-term sequelae. Astrocyte reactivity plays a pivotal role in driving the neuroinflammatory cascade in response to pathological stimuli from peripheral sources or other cellular components of the CNS. The involvement of astrocyte reactivity in the inflammatory process induced by bacterial infection within the CNS warrants further investigation. In this study, we observed an astrocyte reaction likely mediated by brain microvascular endothelial cells (BMEC) during meningitic *E. coli* infection in both a mouse model and a BMEC-astrocyte coculture system. Through label-free quantitative proteomics analysis of the BMEC secretome, we identified CK2 as a potential trigger for astrocyte reactivity. Inhibition of CK2 attenuated the reaction of hippocampal astrocytes in *E. coli* meningitis. Furthermore, we demonstrated that CK2 enhances NF-κB activation *via* its interaction with myosin 9, thereby increasing astrocyte reactivity and the inflammatory response both *in vivo* and *in vitro*. By conditionally knocking out CK2β in microvessel, we blocked CK2 secretion, resulting in reduced astrocyte reactivity and neuroinflammation during the early stages of infection. Compared to wild-type mice, *CK2βVas*^*-/-*^ mice exhibited a significantly higher survival rate. Collectively, our findings highlight the essential role of endothelial-glial communication mediated by CK2 interaction with myosin 9 in activating the downstream NF-κB pathway, contributing to astrocyte reactivity and neuroinflammation. These results provide novel insights into the treatment of CNS inflammation caused by bacterial blood-borne infections.

## Author summary

*Escherichia coli* is the predominant causative agent of gram-negative bacterial meningitis in infants and children. Meningitis caused by *E. coli* is associated with significant mortality and high prevalence of neurological sequelae among survivors. Neuroinflammation, driven by the synergistic interactions of multiple cell types including astrocytes, microglia, and neutrophils, represents a critical pathological process in *E. coli*-induced meningitis; however, the underlying mechanisms remain incompletely understood. This study elucidates a novel mechanism by which meningitic *E. coli* triggers neuroinflammatory responses. During *E. coli* infection, CK2 secreted by brain microvascular endothelial cells (BMEC) interacts with myosin 9 in astrocytes via its β subunit, thereby inducing downstream NF-κB activation and early astrocyte responses. Conditional knockout of CK2β in BMEC inhibits CK2 secretion, consequently alleviating astrocyte activation and neuroinflammation induced by meningitic *E. coli*. Our findings provide new insights into the development of therapeutic strategies for neuroinflammation arising from *E. coli* meningitis.

## Introduction

Bacterial meningitis, defined as inflammation of the meninges and brain parenchyma caused by pathogenic bacteria, is associated with a considerable mortality rate and a high incidence of neurological sequelae in survivors [1,2]. *Escherichia coli* (*E. coli*) is the primary causative agent of gram-negative bacterial meningitis in infants and children [3]. In the majority of cases, meningitic *E. coli* disseminates hematogenously and ultimately traverses the blood-brain barrier (BBB) into the central nervous system (CNS), accompanied by pronounced inflammatory processes [4]. Inflammation within the CNS is considered a key pathological process of *E. coli* meningitis, whereas the mechanisms by which meningitic *E. coli* causes neuroinflammatory responses are incompletely understood. The interaction between bacteria and brain microvascular endothelial cells (BMEC) in the blood-brain barrier (BBB) is a necessary precursor to the development of *E. coli* meningitis [5]. However, whether the stimulated BMEC is involved in the neuroinflammation caused by meningitic *E. coli* is unclear.

Astrocytes, the main glial cells in the CNS, regulate normal brain functions and are key downstream effectors of the inflammatory response that occurs in the CNS [6,7]. Astrocyte reaction, also known as reactive astrogliosis, represents a spectrum of astrocytic molecular, cellular, and functional alterations that occur in response to pathological events, exhibiting a pivotal influence on neuroinflammation [8]. The induction of astrocyte reactivity may be associated with pathogenic processes, including inflammatory responses and neuronal damage, in neurodegenerative diseases such as Alzheimer’s disease and Parkinson’s disease [9,10]. Whether the astrocyte reaction is involved in the inflammatory process in the CNS caused by bacterial infection needs to be further studied. It has been demonstrated that pro-inflammatory microglia can induce a neurotoxic phenotype in astrocytes [11]. Communication between BMEC and astrocytes has also proved to be essential for maintaining CNS homeostasis in normal physiological processes and modulating the inflammatory response under pathological conditions [12]. Recently, more advanced and reliable studies have shown that BMEC is the direct trigger of reactive astrogliosis [13,14].

Casein kinase 2 (CK2) is a highly conserved serine-threonine kinase that is involved in the phosphorylation of at least 300 candidate substrates in all eukaryotic cells [15,16]. The activity of CK2 is contingent upon its tetrameric structure, which comprises two regulatory (β) and two catalytic subunits (α or α′) in a homozygous or heterozygous composition (α2β2; αα′β2; α′2β2) [17]. CK2 has been demonstrated to regulate the inflammatory response process by phosphorylating and modulating the actions of several key transcription factors, including NF-κB, STAT1, cAMP, and C/EBP [18]. Moreover, CK2 plays a role as an ecto-kinase in several extracellular processes, including the phosphorylation of diverse extracellular matrix proteins, receptor proteins, and complement molecules [19]. However, few studies have examined the potential involvement of ecto-CK2 in intracellular processes.

In the present study, we observed that astrocytes underwent a reaction and inflammatory response induced by BMEC during meningitic *E. coli* infection. CK2 was found to be significantly upregulated in the supernatant of BMEC following bacterial stimulation. Moreover, our results suggest that CK2-induced activation of myosin 9-NF-κB signaling may potentially regulate reactive astrogliosis and inflammation. These findings indicate that extracellular CK2 derived from BMEC plays a role in the pro-inflammatory transcriptional reprogramming of reactive astrocytes. Consequently, this discovery provides a promising avenue for developing a therapeutic approach for neuroinflammation resulting from meningitic *E. coli* infection.

## Methods

### Ethics statement

The current study followed the guidelines established by the China Regulations for the Administration of Affairs Concerning Experimental Animals (1988) and Regulations for the Administration of Affairs Concerning Experimental Animals in Hubei Province (2005). All procedures and handling techniques were approved by The Scientific Ethic Committee of Huazhong Agricultural University (Animal Welfare Assurance No. HZAUMO-2023–0289 and No. HZAUMO-2025–0156). All efforts were made to ensure the ethical treatment of all experimental animals in this study and to minimize their suffering.

### Cell culture and bacterial strains

Human brain microvascular endothelial cells (hBMEC) were purchased from ScienCell (Carlsbad, CA, USA). hBMEC were cultured in RPMI 1640 medium supplemented with 10% fetal bovine serum and penicillin-streptomycin (100 U/mL). The human astrocytoma cell line U251 and HEK293T (ATCC CRL-3216) were cultured in Dulbecco’s modified Eagle’s medium (DMEM) supplemented with 10% fetal bovine serum. All the cells were cultured in 37°C incubators under 5% CO_2_ until reaching monolayer confluence. The meningitic *E. coli* strain PCN033, a highly virulent isolate, was initially isolated from cerebrospinal fluid of porcine in China in 2006 [20].

### Animals and infection

Specific-pathogen-free (SPF) C57BL/6 mice were purchased from the Laboratory Animal Services Centre of Huazhong Agricultural University (Hubei, China). Vascular CK2β-specific knockout C57BL/6J mice (*CK2βVas*^*-/-*^) used in this study were obtained by crossing CK2βflox/flox mice (Cat No. S-CKO-01917, Cyagen Biosciences Inc, Jiangsu, China) with Cdh5-Cre mice (Cat No. 001023, Cyagen Biosciences Inc, Jiangsu, China) [21]. The CK2βflox/flox mice were obtained through genetic editing technology, with a loxP site inserted into the intron 2 and intron 3 of the Csnk2b gene on the 17th chromosome of the C57BL/6J mouse fertilized egg. When Cre enzyme is present, the Csnk2b gene can be knocked out by deleting the exon 3. The Cdh5-Cre mice were obtained by inserting the Cre element downstream of the promoter of the Cdh5 gene, which encodes the vascular endothelial cadherin, in the fertilized eggs of C57BL/6N mice. *Cdh5-Cre*-negative *CK2β*^*flox/flox*^ littermates were used as the control group. These mice can specifically express the Cre enzyme in the blood vessels and the conditional knockout was induced by estrogen analogues (tamoxifen). Tamoxifen (MCE, HY-13757A) was dissolved in a mixture of ethanol and corn oil (Solarbio, 1:9 by volume) at a concentration of 25 mg/mL concentration. It was administered through intraperitoneal injection at 2 weeks of age, with a dose of 40 mg/kg body weight every day for a duration of 7 days. Male and female mice 3 w of age were used for experiments.

**Fig 1:**
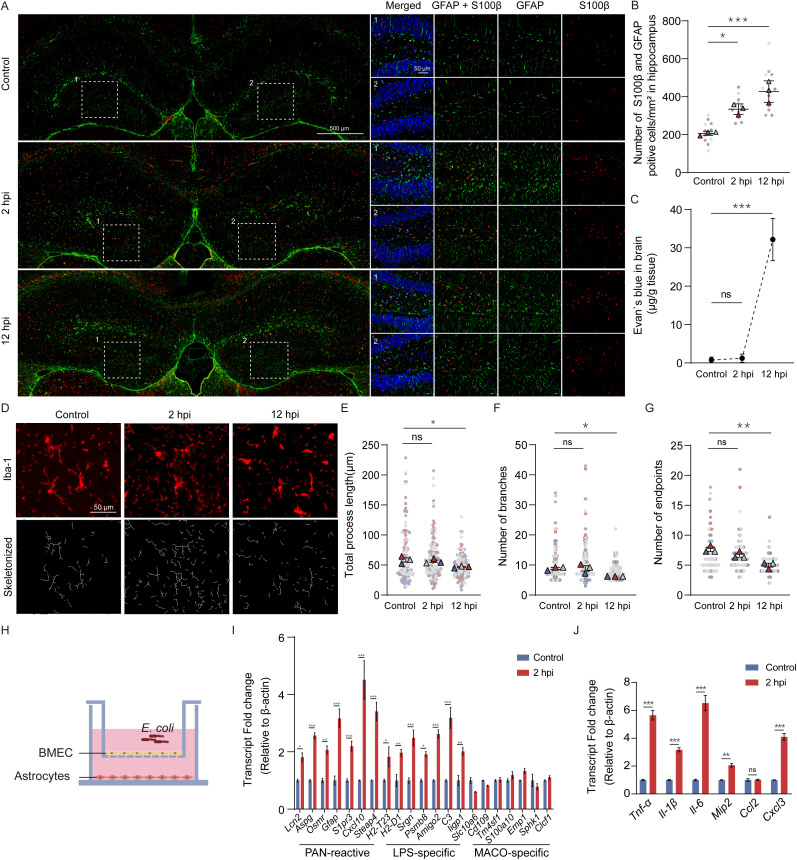
Meningitic *E. coli*-stimulated BMEC induces astrocyte reaction. (A) Representative immunofluorescence of GFAP (green) and S100β (red) in the hippocampal region of control, 2 hpi and 12 hpi group. Scale bar, 500 μm and 50 μm. (B) Number of S100β and GFAP double-positive cells was quantified in 1 mm2 of hippocampal region per section (n = 3 mice per group. For each mouse, four random zones per section were analyzed. The median value from these four fields in all three mice was used for statistical analysis). (C) Quantification of the Evans blue extravasation in the mouse brain (n = 3 mice per group). (D) Representative immunofluorescence of Iba1 (red) and the skeletonized images in the hippocampal region of control, 2 hpi and 12 hpi group. Scale bar = 50 μm. (E-G) Total process length, branches number and endpoints number of the top 10 Iba-1 positive microglia in branches number in 4 fixed regions within the hippocampal region of control, 2 hpi and 12 hpi group (n = 3 mice per group. The median value from these four regions in all three mice was used for statistical analysis). (H) Schematic diagram of the BMEC-astrocyte coculture model. (I) The mouse primary brain microvascular endothelial cells in upper chamber were challenged for 2 h with 10 MOI meningitic *E. coli* or left untreated; mRNA fold changes of astrocytic pan-reactive markers, LPS-specific reactive markers and MCAO-specific reactive markers in mouse primary astrocytes. n = 3. (J) mRNA fold changes of pro-inflammatory factors in mouse primary astrocytes at 2 hpi. n = 3. Data are shown as mean ± SEM. One-way ANOVA (B, C, E, F, G) followed by Tukey’s multiple comparison and Two-way ANOVA (I, J) followed by Bonferroni’s multiple comparison tests; *p < 0.033, ** p < 0.002, *** p < 0.001, ns, not significant.

The meningitic *E. coli* infection model was established as follows. Mice in the infection group were challenged with PCN033 via intravenous tail injection at a density of 1 × 10^7^ CFU. Mice in the control group were injected with isometric PBS. Then, the mice were euthanized with 30% CO_2_ inhalation in a dark chamber at 2 hpi and 12 hpi (Figs 1A–1G; S1A–S1F, S1H–S1K)/ at 2 hpi only (Figs 3G, 3H; 6A, 6D–6G; and S5E–S5H), and their brains were collected for mRNA/protein sample isolation and IF assays [22].

**Fig 2:**
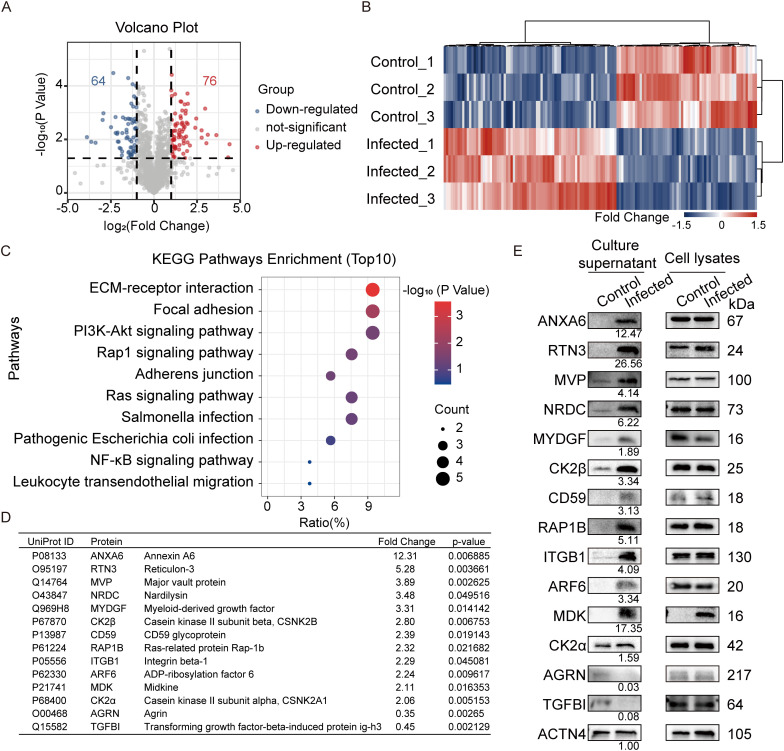
Differential secretome profiling of BMEC in response to meningitic *E. coli* infection. (A) Volcano plot comparing DEPs from the hBMEC culture supernatants of the control/infected groups, n = 3. (B) Heatmap visualization shows unsupervised clustering of differentially expressed proteins (DEPs) from the hBMEC culture supernatants of the control/infected groups. The expression profiles are displayed with three biologically independent samples in each group. Blue represents lower expression, and red represents higher expression. (C) KEGG analysis of the top 10 enriched pathways of the secreted DEPs. (D) Fold changes and p-values of 14 randomly selected DEPs. (E) Western blot verification of the expression of the randomly selected DEPs in the cell culture supernatant and lysate of fresh samples; ACTN4 (fold change of 1.01 in secretome result) here was used as a loading control for the supernatant sample, and fold changes of DEPs (presented as infection/control ratios under the bands of infected group) in culture supernatant were analyzed by Image J. n = 3. Data are shown as mean ± SEM.

**Fig 3:**
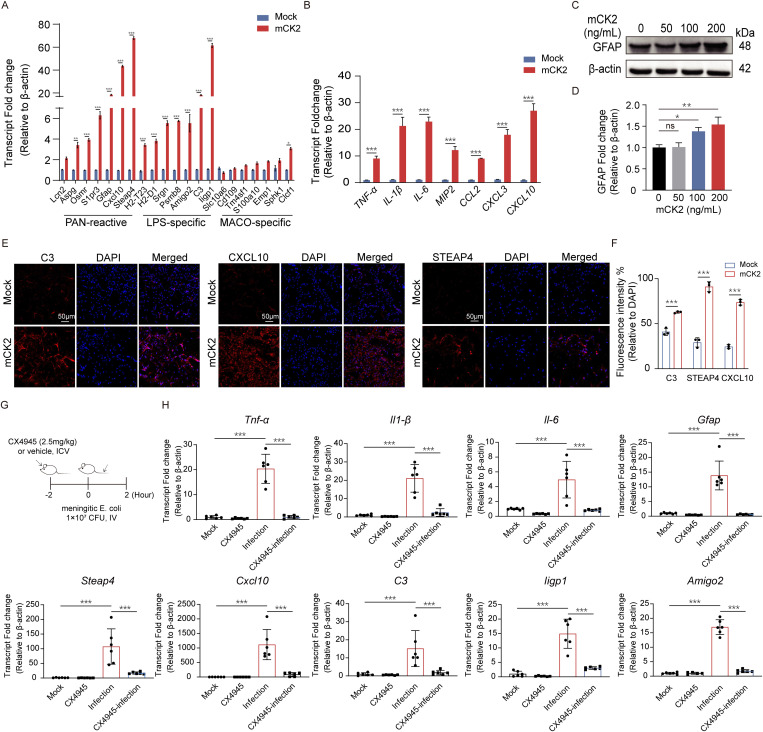
CK2 induces astrocyte reaction *in vitro* and *in vivo.* 100 ng/mL recombinant mCK2 was added to the cell culture supernatant of mouse primary astrocytes for 2 h, the mRNA fold change in the expression of (A) astrocytic pan-reactive markers, LPS-specific reactive markers in mouse primary astrocytes, and (B) pro-inflammatory factors in mouse primary astrocytes, n = 3. (C-D) Protein levels and fold changes of GFAP in mouse primary astrocytes treated with 0, 50, 100 and 200 ng/mL mCK2 for 2 h, n = 3. (E-F) Immunofluorescence and quantification of C3, CXCL10 and STEAP4 (all in red) in mouse primary astrocytes after 200 ng/mL mCK2 treatment. Scale bar, 50 μm. (G) Schematic illustration of mice pretreated with CX4945 (2.5 mg/kg) or vehicle (DMSO) for 2 h via intraventricular injection, followed by intravenous tail injection of meningitic *E. coli* (1 × 10^7^ CFU) for 2 h. (H) mRNA fold changes of reactive astrocyte markers and pro-inflammatory factors in the hippocampus of mice. n = 6. Data are shown as mean ± SEM. Two-way ANOVA (A, B, F) followed by Bonferroni’s multiple comparison test and One-way ANOVA (D, H) followed by Tukey’s multiple comparison; *p < 0.033, **p < 0.002, *** p < 0.001.

**Fig 4:**
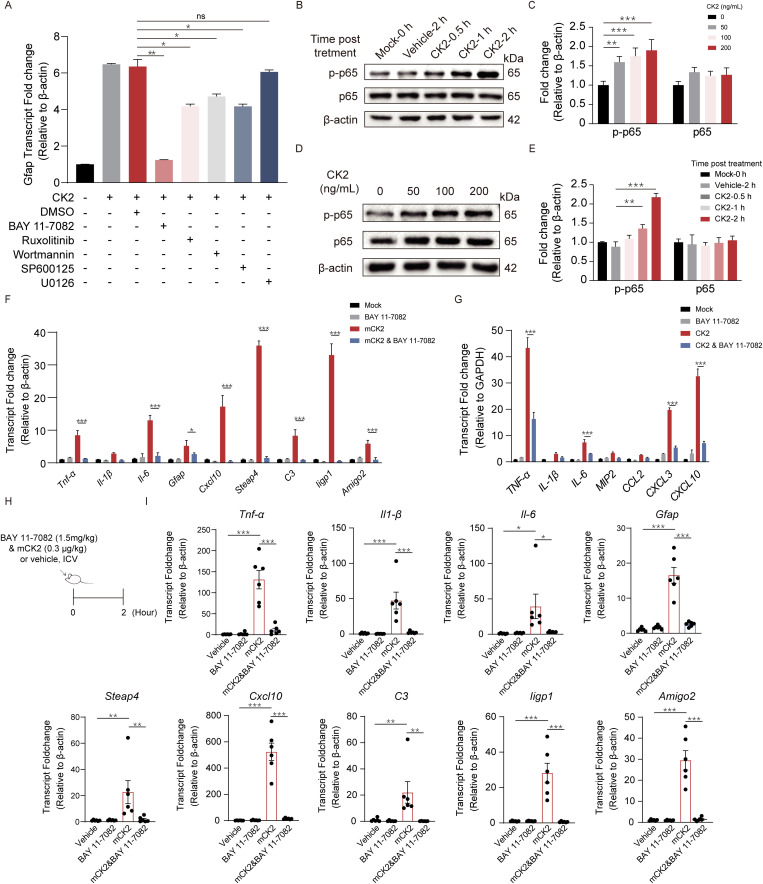
Extracellular CK2 induces astrocyte reaction via the NF-κB signaling pathway. (A) mRNA fold change of *Gfap* in mouse primary astrocytes pretreated with 10 μM BAY 11-7082 (NF-κB inhibitor), Ruxolitinib (JAK/STAT inhibitor), SP600125 (MAPK-JNK inhibitor), Wortmannin (PI3K-Akt inhibitor), U0126 (MAPK-ERK inhibitor) or DMSO (vehicle) for 2 h before mCK2 (100 ng/mL) stimulation. n = 3. Protein levels of phosphorylated p65 and p65 and fold change analysis in mouse primary astrocytes treated with recombinant CK2 in (B-C) a time and (D-E) a concentration gradient. The CK2 reconstitution buffer was used as the vehicle. n = 3. (F) mRNA fold changes of reactive astrocyte markers and pro-inflammatory factors in mouse primary astrocytes pretreated with 10 μM BAY 11-7082 or isometric DMSO for 2 h before 100 ng/mL mCK2 stimulation for 2 h. n = 3. (G) mRNA fold changes of pro-inflammatory factors in U251 pretreated with 10 μM BAY 11-7082 or isometric DMSO for 2 h before 100 ng/mL CK2 stimulation for 2 h. n = 3. (H) Schematic illustration of mice treated with 1.5 mg/kg BAY 11-7082 (or vehicle, DMSO) and 0.3 μg/kg mCK2 simultaneously for 3 h. (I) mRNA fold change of reactive astrocyte markers and pro-inflammatory factors in the hippocampus of the mice. n = 6. Data are shown as mean ± SEM. One-way ANOVA (A, I) followed by Tukey’s multiple comparison and Two-way ANOVA (C, E, F, G) followed by Bonferroni’s multiple comparison test. *p < 0.033, **p < 0.002, ***p < 0.001, ns, not significant.

**Fig 5:**
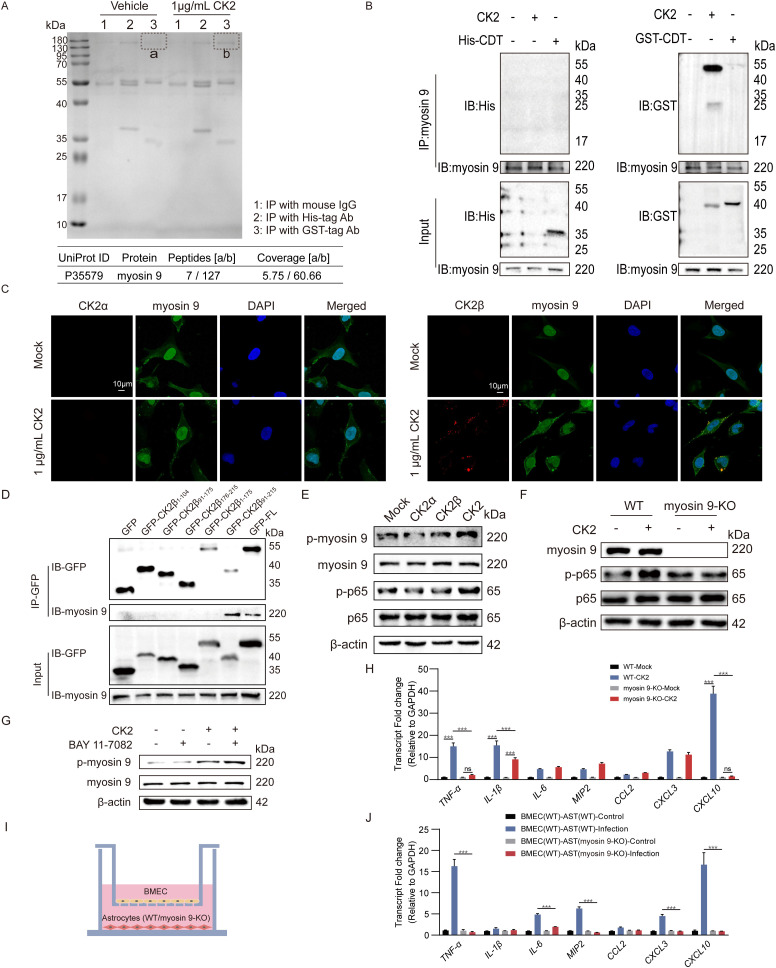
Extracellular CK2 interacts with myosin 9 in astrocytes. (A) Analysis of endogenous astrocytic proteins associated with extracellular CK2 by immunoprecipitation and LC-MS/MS. A total of 1 μg/mL recombinant CK2 consisting of His-tagged CK2α & GST-tagged CK2β was added into the cell culture supernatant of U251 cultured in 10 cm dishes for 3 h. The cells were subsequently lysed and centrifuged, the clarified cell supernatant was immunoprecipitated with a mouse monoclonal antibody for His and GST tags (2 μg per 500 μg of cell lysates), and mouse IgG was used as a negative control. Specific bands in lane 3 in the vehicle group and CK2 group (a and b) were then identified by LC-MS/MS. Relevant information on the top-ranked protein myosin 9 is shown. (B) U251 cells were incubated with 1 μg/mL recombinant CK2 or His-tagged/GST-tagged CDT (as negative controls) for 2 h. Then, the cell lysates were immunoprecipitated with an anti-myosin 9 antibody and immunoblotted with an anti-His antibody or anti-GST antibody, respectively. (C) Immunostaining of His-tagged CK2α (red), GST-tagged CK2β (red) and endogenous myosin 9 (green) in U251 after stimulation with 1 μg/mL CK2 for 2 h. Scale bar, 10 μm. (D) HEK293T cells were transfected with expression vectors encoding GFP or CK2β truncation mutants. The cell lysates were immunoprecipitated with an anti-GFP antibody and immunoblotted with an anti-GFP antibody or anti-myosin 9 antibody. (E) Protein levels of phosphorylated myosin 9, myosin 9, phosphorylated p65 and p65 in mouse primary astrocytes at 30 min after 100 ng/mL CK2α, CK2β and CK2 treatment. n = 3. (F) Protein levels of myosin 9, phosphorylated p65 and p65 in WT and myosin 9-KO U251 cells after 100 ng/mL CK2 treatment for 1 h. CK2 reconstitution buffer was used as the vehicle. n = 3. (H) mRNA fold change of pro-inflammatory genes in WT/ myosin 9-KO U251 cells stimulated with 100 ng/mL CK2 for 2 h. n = 3. (G) Protein levels of phosphorylated myosin 9 and myosin 9 in mouse primary astrocytes pretreated with BAY 11-7082 (NF-κB inhibitor) or vehicle (DMSO) following 100 ng/mL CK2 treatment for 10 min. n = 3. (I) Schematic diagram of the hBMEC (WT) cocultured with U251 (WT) or U251 (myosin 9-KO). (J) mRNA fold change of pro-inflammatory genes in WT/myosin 9-KO U251 within the coculture model under infection conditions. n = 3. Data are shown as mean ± SEM. Two-way ANOVA (H, J) followed by Bonferroni’s multiple comparison test. ***p < 0.001, ns, not significant.

**Fig 6:**
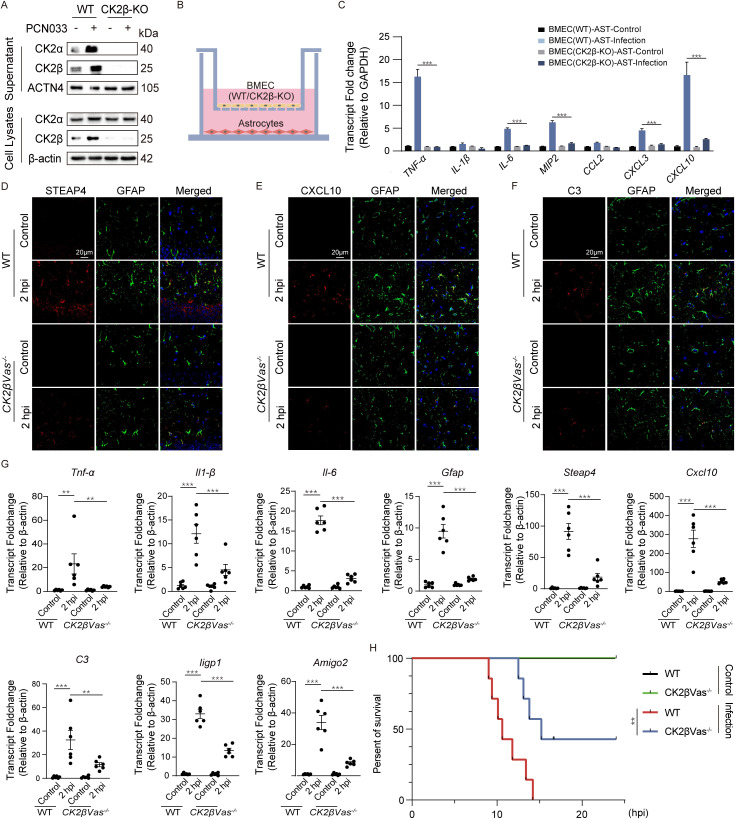
Loss of CK2β in the BMEC attenuates astrocyte reaction induced by the meningitic *E. coli* infection. (A) Protein levels of CK2α and CK2β in supernatants and lysates of WT and CK2β-knockdown hBMEC after meningitic *E. coli* infection (10 MOI for 2 h). n = 3. (B) Schematic diagram of the hBMEC (WT) or hBMEC (CK2β-KO) cocultured with U251 cells. (C) mRNA fold change of pro-inflammatory genes in U251 cells cocultured with WT/CK2β-KO hBMEC under infection condition. n = 3. Immunofluorescence of (D) STEAP4, (E) CXCL10, and (F) C3 (all in red) with GFAP (green) in the hippocampus of WT and *CK2βVas*^*-/-*^ mice post 2 h infection. Scale bar, 20 μm. n = 3. (G) mRNA fold change of reactive astrocyte markers and pro-inflammatory factors in hippocampus of WT and *CK2βVas*^*-/-*^ mice challenged with meningitic *E. coli* (1 × 10^7^ CFU). n = 6. (H) Survival rates of WT and *CK2βVas*^*-/-*^ mice were monitored for 25 h after meningitic *E. coli* infection. n = 7. Data are shown as mean ± SEM. One-way ANOVA (G) followed by Tukey’s multiple comparison and Two-way ANOVA (C) followed by Bonferroni’s multiple comparison test. Log-rank (Mantel-Cox) test (H). ** p < 0.002, *** p < 0.001, ns, not significant.

For stereotaxic injection, mice were anesthetized with isoflurane (1.5%) in pure oxygen using continuous flow via a nose cone to maintain a surgical plane of anesthesia. Sedation was assessed via absence of both the pedal (toe pinch) and tail pinch reflexes, and anesthesia was maintained at a constant rate. After confirming proper sedation, the fur from the top of the scalp to the base of the neck was shaved and mice were fixed on a stereotaxic apparatus with ear bars. After the skull was exposed via a small incision, a small hole was drilled for injection based on coordinates with the bregma. For *in vivo* compound or protein delivery, microinjection was performed at the following stereotaxic coordinates of the bilateral ventricle: anteroposterior (AP), -0.8 mm; mediolateral (ML), ± 1.7 mm; and dorsoventral (DV), -2.5 mm from bregma. The injection was performed via a 10 μL gas phase (tip) microinjector (Shanghai Gaoge Industry and Trade Co., Ltd) over 10 min; the needle was not removed until 10 min after the infusion. All the compounds and proteins were injected at a total volume of 10 μL [23]. After completing injections into both hemispheres, the incision was closed using a stainless steel wound clip and the mice were placed back on the warming pad until they woke up and moved around normally.

### Reagents, antibodies and inhibitors

The NF-κB inhibitor BAY11–7082, the JAK/STAT inhibitor Ruxolitinib, the JNK inhibitor SP600125, the PI3K-Akt inhibitor Wortmannin, the ERK1/2 inhibitor U0126, and the CK2 inhibitor Silmitasertib (CX-4945) were purchased from MedChem Express (Monmouth, USA). Recombinant human casein kinase II (CK2) was purchased from Sigma Aldrich (218701, St. Louis, USA). The nucleic acid dye, 4’-6-diamidino-2-phenylindole (DAPI), was obtained from Solarbio (Beijing, China). All the antibody information was placed in S5 Table.

### Evaluation of BBB permeability by Evans blue

The mice were anesthetized according to the method described in “Animal and infection”. Evans blue (2% wt/vol in PBS, 2 mL/kg) was administered through the tail vein 0.5 h before mice were euthanized. Whereafter, mice were perfused through the left ventricle with 10 mL PBS to remove intravascular albumin-EB. Then half of the brain was weighed and homogenized in 1 mL of PBS, and 0.5 mL homogenization was mixed with 1.5 mL of 50% trichloroacetic acid, followed by vortex mixing for 2 min. After centrifugation at 10,000 × g for 30 min at 4°C, the supernatant was collected. 30 µL of supernatant was mixed with 90 µL 95% ethanol in a clear 96-well plate. The fluorescence intensity was measured at 620 nm excitation/680 nm emission on a microplate fluorescence reader (SPARK 10M, TECAN) and compared against a standard curve (serial dilutions of the stock dye solution in the concentration range of 0, 0.25, 0.5, 1, 5, 20 μg/mL), as described previously [24]. The Evans blue content was calculated and expressed as per gram of brain tissue.

### Preparation of mouse primary astrocyte and endothelial cell cultures

Primary cultures of astrocytes were prepared from neonatal mice, as described previously [25]. Newborn mice (1-2d postnatal) were killed by decapitation, and the brains were removed. After the meninges were removed from the entire brain, the neocortical regions were excised and collected. The tissues were dissociated with 0.25% trypsin at 37°C, and the reaction was terminated with DMEM supplemented with 10% fetal bovine serum and 1% penicillin/streptomycin. The cell culture medium was refreshed 24 h later and changed every 3 days. Experiments were performed on confluent 14-day-old cultures.

Endothelial cells were isolated from adult mice of 6–8-week-old as described previously [26]. The brains were cut along the longitudinal fissure to separate the two hemispheres. The meninges and choroid plexuses were removed by rolling the brain tissues on P8 grade filter paper. Minced brain pieces were forced through sterile needles in an 18G, 20G and 22G needles order. After centrifugation at 800 × g for 8 min at 4°C, the precipitate of the homogenate was digested and pelleted. The myelin was separated by resuspending the pellet in DMEM containing 20% BSA and centrifuging at 4˚C, 1,000 × g for 20 min. In order to separate microvessel from brain cellular contaminants, the pellet was resuspended in 1 ml of ice-cold DMEM and overlay on top of a cold 33% continuous isotonic Percoll gradient and then centrifuged at 4˚C, 1,000 × g for 20 min. The microvessel layer was collected, washed and seeded at a dilution corresponding to one brain per well on 6-well plates previously coated for 4 h with type IV collagen. Microvessel were cultured in high glucose DMEM supplemented with 20% FBS, 1% penicillin/streptomycin, 1 ng/mL basic fibroblast growth factor (bFGF) and 100 μg/mL heparin. The culture medium was replaced every other day using fresh ECs culture medium containing only 4 μg/mL puromycin.

### BMEC-astrocytes transwell coculture and infection assays

The *in vitro* BMEC-astrocyte coculture model was constructed via a 0.4 μm pore size transwell chamber following previously reported methods [27]. In brief, mouse primary endothelial cells or hBMEC were pre-seeded in the upper chamber of transwell inserts (Corning Cellgro, Manassas, VA, USA) at 8 × 10^3^ cells per well, and mouse primary astrocytes or U251 cells were synchronously seeded in a 24-well plate. After 12 h of growth, the transwell inserts were transferred to the 24-well plates, and the coculture medium was changed to DMEM supplemented with 1 ng/mL bFGF (for primary cells) or RPMI 1640 medium (for cell lines). Meningitic *E. coli* infection of coculture model was performed as follows. The confluent cells were starved in serum-free medium for 12 h. The meningitic *E. coli* cultures were resuspended, diluted in the same serum-free medium and added into the transwell inserts at the indicated time points (10 MOI in 100μL final volume). Afterwards, the cells in the lower chamber were washed twice with pre-chilled PBS and collected for the subsequent RNA isolation assay.

### Immunohistochemistry and immunofluorescence

For immunohistochemistry, anesthetized mice (according to the method described in “Animal and infection”) were perfused with PBS transcardially, followed by 4% paraformaldehyde. Then, the mouse brain tissues were fixed with 4% paraformaldehyde at 4°C for 24 h. A series of gradient alcohols, anisole and xylene were used for the tissue dehydration before paraffin embedding: 75% ethanol for 5 min, 85% ethanol for 5 min, 90% ethanol for 5 min, 95% ethanol for 5 min, absolute ethanol twice for 5 min each, anisole for 5 min, xylene twice for 5 min each. Fixed brains were sliced into 5 μm thickness coronal sections using a Leica RM2125 RTS microtome. The sections were placed in a 37°C warm water bath until it is completely flat and then were mounted by being placed in 37 °C heater for 2 h. Sections were deparaffinized by xylene twice for 5 min each and were hydrated with 100% ethanol twice for 5 min each, 95% ethanol twice for 5 min each, and 70% ethanol twice for 5 min each and then rinsed in distilled water. Then, the paraffin sections were heated in citrate buffer (pH 6.0) at 60°C for 10 min. After natural cooling, sections were placed in PBS and washed 3 times. Then, sections were processed with 0.1% Triton X for 5 mins, followed by 5% bovine serum albumin blocking for 1 h at room temperature. After staining with primary antibody (diluted in the blocking buffer) overnight at 4°C. After washing off with PBS, the samples were then incubated with secondary antibodies for 1 h at room temperature. After that, the samples were covered with DAPI (diluted in the blocking buffer) in the dark. For immunofluorescence for cultured cells, cells were washed with PBS at 4°C and fixed with 4% paraformaldehyde for 30 min at room temperature, followed by incubation in blocking and permeabilization buffer containing PBS with 0.1% Triton X-100 and 5% normal bovine serum.

Primary and secondary antibodies are listed in S5 Table. Finally, the cells or sections were stained with the indicated antibodies and DAPI. Images were taken with an LSM 880 confocal microscope (Carl Zeiss AG, Oberkochen, Germany). For quantification, the intensities of the immunopositive cells were measured via Fiji ImageJ software. For S1I Fig, Pearson’s correlation coefficient was computed using Fiji ImageJ software with the Trainable Weka Segmentation and ROI manager to assess the degree of colocalization between albumin and CD31, providing a semi-quantitative indicator of dynamic changes of BBB permeability over time. For Fig 1E–1G, morphological analysis (skeletonization, total process length measurement, and branch number/endpoints counting) was performed by plugins AnalyzeSkeleton (2D/3D) of Fiji ImageJ software according to the previous studies [28].

### Sample preparation, data acquisition, and analysis of the BMEC secretome

The mass spectrometry data of the BMEC secretome have been deposited in the ProteomeXchange Consortium via the PRIDE partner repository with the dataset identifier PXD021427. Primary hBMEC were divided into control and infected groups and were starved in a serum-free medium (1:1 mixture of Ham’s F-12 and M-199) for 16 h. The medium of the control group was replaced with the fresh serum-free medium when the other group was infected with PCN033 for 3 h (MOI = 10) at 37°C. Cell culture supernatants from the infected and control groups were obtained and centrifuged at 4,000 × g for 15 min at 4°C. The insoluble debris was removed, and the supernatant was centrifuged at 15,000 × g for 2 h at 4°C. The protein concentration in the supernatant was measured via a BCA protein assay kit (Beyotime, China) and then reduced and alkylated with 10 mM Tris-(2-carboxyethyl) phosphine and 40 mM chloroacetamide at 95°C for 5 min. Alkylated proteins were diluted fivefold with 50 mM triethylammonium bicarbonate and digested with Lys-C at a 1:100 (wt/wt) enzyme-to-protein ratio for 3 h at 37°C. Trypsin was added at a final 1:50 (wt/wt) enzyme-to-protein ratio overnight digestion at 37°C. The digested peptides were acidified with trifluoroacetic acid to a final concentration of 0.5% trifluoroacetic acid, and 250 μL of ethyl acetate was added to 250 μL of the digested solution. The mixture was shaken for 2 min and then centrifuged at 20,000 × g for 2 min to obtain aqueous and organic phases. The aqueous phase was collected and desalted via a 100 mg Sep-Pak C18 column. The in-house-constructed immobilized metal ion affinity chromatography (IMAC) tip was made by capping the end with a 20 μm polypropylene frit disk. Peptides were dissolved in 4 μL of 0.3% formic acid with 3% (vol/vol) acetonitrile and injected into an EasynLC 1000. Peptides were separated on a 45-cm in-house packed column (360 μm OD × 75 μm inner diameter) containing C18 resin (2.2 μm, 100 Å) with a 30-cm column heater, and the temperature was set at 50°C. The mobile phase buffer consisted of 0.1% formic acid in ultrapure water (buffer A) with an elution buffer of 0.1% formic acid in 80% (vol/vol) acetonitrile (buffer B), which was run with a linear 45- or 60-min gradient of 6–30% buffer B at a flow rate of 250 nL/min. The EasynLC 1000 was coupled online with a hybrid high-resolution LTQ-Orbitrap Velos Pro mass spectrometer. The mass spectrometer was operated in data-dependent mode, in which a full-scan MS (from m/z 350–1,500 with a resolution of 30,000 at m/z 400) was used, followed by MS/MS on the 10 most intense ions (normalized collision energy, 30%; automatic gain control 3E4; maximum injection time, 100 ms). For the raw MS/MS data, the intensities of the peptides were extracted via the MaxQuant search engine with Label-Free Quantitation (LFQ), and the analysis was processed via Andromeda. The tandem mass spectra results were subsequently matched with the Human UniProt database. The relative protein abundances were presented as the infection/control ratios. The statistical significance of the difference between the control and infection group was analyzed by t-test followed by Bonferroni’s multiple comparisons. Proteins whose expression changed more than 2 times or less than 0.5 times and whose p-value was less than 0.05 were regarded as differentially expressed proteins. After removing the proteins that were marked by UniProt for their nuclear localization, SignalP 6.0 (https://services.healthtech.dtu.dk/services/SignalP-6.0/) was used to predict whether the remaining proteins carried signal peptides, and SecretomeP 2.0 (https://services.healthtech.dtu.dk/services/SecretomeP-2.0/) was used to predict non-classical secretory proteins (NN-SCORE > 0.6).

### RNA extraction and qRT-PCR

The total RNA from cultured cells or tissue isolated with TRIzol reagent was reverse transcribed via HiScript II Q RT SuperMix reverse transcriptase (Takara, Japan). Quantitative real-time PCR (qRT-PCR) was performed on a QuantStudio 3 real-time PCR system (Thermo) with 2 × M5 HiPer SYBR Premix EsTaq (Mei5bio, Beijing, China) according to the manufacturer’s instructions. The primers used for qRT-PCR are shown in S1 Table. Each sample’s abundance of individual mRNA transcripts was assayed in triplicate and normalized to the GAPDH (for the human sample) or β-actin (for the mouse sample) mRNA level via the 2^-ΔΔCT^method.

### Western blot analysis

The cells and homogenized tissues were lysed in ice-cold cell lysis buffer or RIPA buffer (Beyotime, Shanghai, China), and then the samples enriched with 1 × loading buffer were heated to 100°C for 10 min. The protein samples were separated via SDS-PAGE and transferred to polyvinylidene difluoride membranes. The membranes were blocked with 5% bovine serum albumin in TBST for 2.5 h at room temperature and incubated with specific primary antibodies diluted in the blocking buffer overnight at 4°C. The membranes were then probed with the corresponding peroxidase-conjugated secondary antibody for 1.5 h at room temperature. Then the membranes were washed with TBST three times and developed with Clarity Western ECL Substrate (Bio-Rad), the results were visualized via the ChemiDoc XRS1 imaging system (Bio-Rad Laboratories). Primary and secondary antibodies used in western blot are listed in S5 Table. The quantitative analysis of bands was assessed using ImageJ, and β-actin (as well as ACTN4 in the cell culture supernatant protein sample in Figs 2E and 6A) was used as the internal reference protein. Uncropped versions of the blots are shown in S1 Data.

### Recombinant CK2 expression and purification

Human CK2α/CK2β, as well as mouse CK2α/CK2β (mCK2α/mCK2β), were expressed in a prokaryotic expression system as described below. Human *CSNK2A1* and *CSNK2B*were amplified from hBMEC cDNA, and mouse *Csnk2a1* and *Csnk2b* were amplified from the cDNA of a mouse brain microvascular endothelial cell line (bEND.3, ATCC CRL-2299), the primer used in the amplification are listed in Supplementary S1 Table. His-tagged CK2α and mCK2α, as well as GST-tagged CK2β and mCK2β, were then separately overexpressed in *E. coli* BL21 (DE3) after being subcloned and inserted into pET-28a and pGEX-6p-1. The soluble fraction of the bacterial lysates was purified with Ni-NTA His-Tag Purification Agarose (MCE) or Glutathione Sepharose (GE) according to the manufacturer’s instructions. Edulcoration and concentration of the purified proteins were performed with 10 kDa Ultra Centrifugal Filters (Millipore, UFC901024). The endotoxin of the purified proteins was removed by Pierce High-Capacity Endotoxin Removal Resin (Thermo, No.88276). Finally, the purified proteins were mixed with stock buffer (20 mM MOPS, pH 7.4, 1 mM EDTA, 0.1 mM Triton X-100, 0.3% 2-mercaptoethanol, and 50% glycerol) for storage at −80°C. The α and β subunits were reconstituted into the holoenzyme *in vitro* as described previously [29]. Equimolar quantities of each subunit (0.1 nmoL) were incubated for 1 h at 30°C in 200 μL of reconstitution buffer (50 mM Tris-HCl pH 7.0, 150 mM NaCl, 0.1% BSA).

The enzyme activity of α subunits and holoenzymes is reflected by ATP consumption. Briefly, astrocytes cultured in 10 cm dishes were lysed with reconstitution buffer, and the lysate was collected and centrifuged at 10,000 × g for 3 min. For each of the three parallel wells in a 96-well opaque plate, 50 μL of supernatant was incubated with 50 ng of CK2α, mCK2α, CK2 or mCK2 for 10 min at 37 °C. BSA was used as a negative control. The ATP content was subsequently detected via an Enhanced ATP Assay Kit according to the manufacturer’s instructions (Beyotime, Shanghai, China).

The cytotoxicity of CK2 subunits and holoenzymes was assessed by measuring cell viability with a Cell Counting Kit-8 according to the manufacturer’s instructions (Beyotime, Shanghai, China). Astrocytes were seeded and grown in 96-well plates until they reached monolayer confluence. mCK2α, CK2α, mCK2β, CK2β, mCK2 and CK2 (400 ng/mL) were added to the plates for 1 h and five parallel wells were used for each treatment.

### Immunoprecipitation and LC-MS/MS analysis

After CK2 incubation, cellular samples were obtained via lysis buffer for western blot and IP and were subsequently centrifuged at 4°C for 15 min at 15,000 × g to remove insoluble cell debris. The clarified cell lysates (500 μg) were incubated with 2 μg indicated primary antibodies overnight at 4°C, and 25 μL protein A/G agarose (Beyotime, China) was then added for 2 h of incubation at room temperature. After five washes with PBST, the immunoprecipitates were resuspended in 1 × loading buffer and boiled for 5 min. Then, the protein samples were separated by SDS-PAGE. The differential protein bands from the GST-tag antibody-immunoprecipitated samples were identified via LC-MS/MS, performed using a nanoLC-LTQ-Orbitrap XL mass spectrometer (Thermo, San Jose, USA) coupled with an Eksigent nano-LC 1D plus HPLC system at APTBIO (Shanghai, China). Tryptic peptides were fully enzymatically digested and ionized via nano electrospray ionization. The data were analyzed via a full-scan mass spectrometer (300–1800 m/z). Finally, Proteome Discoverer (version 1.4.0.288, Thermo Scientific) was used to analyze the MS data.

### Gene transfection, RNAi, and generation of knockout cell lines

The transfection of siRNA or DNA was performed via jetPRIME transfection reagent (Polyplus, France) according to the manufacturer’s protocol. Human *CSNK2B* siRNA and negative control were synthesized by JTS Scientific, and the knockdown efficiency was determined at 48 h post-transfection via western blot analysis. The sgRNA sequences targeting human *CSNK2B* and the sgRNA targeting human *MYH9* were predicted via the online CRISPR/Cas9 design tool (https://www.genscript.com.cn/gRNA-design-tool.html) and inserted into individually cloned into a pYSY-SpCas9-sgRNA-Puro plasmid. The recombinant sgRNA expression plasmids were cotransfected into cells for 24 h. Then, the positive-transfected cells were selected by fresh medium containing 1 μg/mL puromycin for another 24 h. Surviving cells were transferred into 96-well plates with limiting dilution and incubated until a single-cell clone formed. Positive single-cell clones were validated via DNA sequencing and immunoblotting analyses.

### Statistical analysis

The results are expressed as mean ± SEM, for datasets that do not follow a normal distribution, median was used for statistical analysis, and all the statistical analyses are described in the figure legends. Brown-Forsythe test was used to conduct a homogeneity of variance test on the data. In all *vivo* experiments, we prepared two sections of each mouse and took two photos of each section. Two randomly selected photos from different brain slices of each mouse were used for immunohistochemistry analysis. Especially, 4 regions of interest (S1G Fig) per mouse were analyzed in Figs 1B, 1E–1G and S1I Data in Figs 1B, 1E–1G and S1I were presented in the form of significance of the differences between each group was analyzed by one-way ANOVA followed by Tukey’s multiple comparison. In all the figures of subculture cells, “n = 3” represents three separate wells of distinct cell cultures. In the case of using primary cells, “n = 3” refers to three independent cultures obtained from the same cell isolation. For normally distributed datasets, data was analyzed by one-way ANOVA followed by Tukey’s multiple comparison, two-way ANOVA followed by Bonferroni’s multiple comparison test or Log-rank (Mantel-Cox) test (Fig 6H). The statistical analyses of all data were performed with GraphPad Prism, version 8.0 (GraphPad Software Inc., La Jolla, CA, USA). p < 0.033 (*) was considered statistically significant, and p < 0.002 (**), as well as p < 0.001 (***), were considered extremely significant.

## Results

### Crosstalk with BMEC facilitates the inflammatory response of astrocytes during meningitic *E. coli* infection

As two major cell types involved in maintaining homeostasis within the CNS, astrocytes and microglia also play an indispensable role in neuroinflammation [30,31]. The immunoreactivity of glial fibrillary acidic protein (GFAP) and S100β, markers of astrogliosis, were predominantly elevated in the hippocampal and internal capsule regions as the duration of infection increased (S1A Fig). While GFAP immunoreactivity levels in both regions were significantly upregulated at 12 h post infection (hpi), unlike in the internal capsule, GFAP levels in the hippocampal region were already significantly enhanced as early as 2 hpi (S1B, S1C Fig). The number of S100-positive cells also increased during the infection (S1D–S1F Fig). More importantly, the number of S100β and GFAP double-positive cells significantly increased at 2 hpi and further rose at 12 hpi in the hippocampal region (Fig 1A and 1B). To ascertain whether the observed astrogliosis could be attributed to the stimulation of factors such as pathogens and virulence proteins leading to peripheral blood bacterial-induced disruption of the BBB, we investigated the dynamic changes in BBB integrity following meningitic *E. coli* infection. Mice were intravenously injected with Evans blue 0.5 h prior to each analysis time point. Compared with the control group, no significant difference in BBB permeability was observed at 2 hpi. However, severe BBB disruption was detected at 12 hpi (Fig 1C). To quantitatively evaluate the extent of albumin extravasation, we utilized Pearson’s correlation coefficient to measure the colocalization of albumin with the brain microvessel marker CD31. A higher Pearson’s coefficient in the control and 2 hpi groups indicates strong colocalization, suggesting that albumin predominantly remains within the microvessel. Conversely, a lower Pearson’s coefficient in the 12 hpi group reflects weaker colocalization, indicating the extravasation of albumin from microvessel. (S1H, S1I Fig). Microglia are widely recognized as key instigators of astrogliosis. To investigate their potential involvement in astrogliosis during the early stage of infection, we performed a detailed morphological analysis of microglia in four fixed regions within the hippocampal region [32]. Compared to the control group, microglia at 12 hpi exhibited a significant reduction in total process length, branch number, and endpoints number, indicative of heightened microglial activation. In contrast, no substantial changes were observed in microglia at 2 hpi (Fig 1D–1G).

Given the critical role of brain microvascular endothelial cells (BMEC) in the blood-brain barrier (BBB) during the progression of *E. coli* meningitis, as well as the close interaction between BMEC and astrocytes, we investigated whether BMEC contributes to the astrocyte response at the early stage of this process. To explore this hypothesis, an *in vitro* coculture system was established to mimic the cell crosstalk between BMEC and astrocytes (Fig 1H). In this model, primary mouse brain microvascular endothelial cells were cultured in transwell inserts, while primary mouse astrocytes were maintained in culture plates. Both cell types shared the same culture medium but remained physically separated. The endothelial cells within the transwell inserts were infected with meningitic *E. coli* PCN033 (MOI = 10) for 2 h. Subsequently, the transcriptional profiles of genes previously identified as pan-reactive markers of astrocytic activation (*Lcn2*, *Aspg*, *Osmr*, *S1pr3*, *Gfap*, *Cxcl10* and *Steap4*), LPS (Lipopolysaccharide)-specific reactive markers (*H2-T23*, *H2-D1*, *Srgn*, *Psmb8*, *Amigo2*, *C3* and *Iigp1*), and MCAO (Middle cerebral artery occluding)-specific reactive markers (*Slc10a6*, *CD109*, *Tm4sf1*, *S100a10*, *Emp1*, *Sphk1,* and *Clcf1*) were analyzed [33]. In comparison to the control group, the results revealed a significant increase in mRNA levels for all 7 pan-reactive markers and 7 LPS-specific reactive markers, while no significant changes were observed in the mRNA levels of MCAO-specific reactive markers. This suggests that astrocytes in the lower chamber may undergo an LPS-specific reaction (Fig 1I). Additionally, we detected transcriptional upregulation of seven pro-inflammatory-related genes (*Tnf-α*, *Il-1β*, *Il-6*, *Mip2*, *Ccl2*, *Cxcl3*) in mouse primary astrocytes, which was further validated in hBMEC-U251 coculture model (Figs 1J and S1L). Based on these findings, we propose that the crosstalk between BMEC and astrocytes plays a critical role in astrocyte reactions and neuroinflammation during meningitic *E. coli* infection.

### Differential secretion proteomics of hBMEC in response to infection

To further investigate potential BMEC regulatory molecules involved in astrocyte reaction, we collected culture supernatants from meningitic *E. coli*-infected as well as uninfected hBMEC for label-free quantitative proteomics analysis. A total of 140 proteins were identified as significant differential expression (fold change >2, p-value <0.05) among the 1680 common proteins identified from the cell culture supernatant. Among these proteins, 76 were upregulated and 64 were downregulated in the infection samples compared to the control samples (Fig 2A). The relative abundance of differentially expressed proteins (DEPs) in three biological replicates for each group is presented in a heatmap (Fig 2B). Furthermore, the DEPs were screened using the secreted protein analysis software SignalP and SecretomeP, resulting in the identification of a total of 61 possible secreted proteins (non-classical secretory proteins and classical secretory proteins carrying signal peptides, S2 Table). A Kyoto Encyclopedia of Genes and Genomes (KEGG) pathway enrichment analysis demonstrated that these secreted DEPs were predominantly associated with meningitic *E. coli* infection of the BBB (e.g., ECM-receptor interaction, focal adhesion, and adherens junction) and neuroinflammation (e.g., the PI3K-Akt signaling pathway, the Ras signaling pathway, pathogenic *E. coli* infection, and the NF-κB signaling pathway) (Fig 2C, S3 Table). Subsequently, 14 proteins were randomly selected from the 61 secreted DEPs for western blot validation. The selected DEPs included 12 significantly upregulated proteins and 2 significantly downregulated proteins. The fold change of these DEPs in the quantitative proteomics analysis, along with the associated p-values, are presented in Fig 2D. In comparison to the internal reference protein ACTN4, the expression of ANXA6 and the other 11 proteins significantly increased in the culture supernatant of hBMEC, and the expression of AGRN and TGFBI decreased, which aligns with the findings of the quantitative proteomics analysis (Figs 2E and S2). Our research demonstrated that meningitic *E. coli* infection of hBMEC resulted in significant alterations in the secretion proteomic profiles of these cells.

### CK2 contributes to the astrocyte inflammatory response

Since NF-κB represents a pivotal inflammatory signaling pathway and both CK2α and CK2β are enriched within this pathway, we selected to pursue subsequent validation of the biological function of CK2. First, we successfully expressed recombinant human and mouse CK2 proteins. Furthermore, recombinant human and mouse CK2 were verified to be enzymatically active and non-cytotoxic (S3A and S3B Fig). mouse primary astrocytes were subsequently treated with mouse recombinant CK2 (mCK2) at a concentration of 100 ng/mL for 2 h. Real-time PCR results demonstrated that mCK2 stimulation significantly upregulated the expression of astrocytic pan-reactive markers (*Aspg*, *Osmr*, *S1pr3*, *Gfap*, *Cxcl10* and *Steap4*), LPS-specific reactive markers (*H2-T23*, *H2-D1*, *Srgn*, *Psmb8*, *Amigo2*, *C3* and *Iigp1*) and pro-inflammatory factors (*TNF-α*, *IL-1β*, *IL-6*, *MIP2*, *CCL2*, *CXCL3*, and *CXCL10*) (Fig 3A and 3B). The western blot results demonstrated that CK2 protein at concentrations of 50, 100, and 200 ng/mL for 2 h stimulation of mouse primary astrocytes dose-dependently induced a significant upregulation of GFAP, which is an important marker molecule for astrogliosis (Fig 3C and 3D). Furthermore, we investigated the expression of C3, CXCL10, and STEAP4 via immunofluorescence following the administration of recombinant mouse CK2 at a concentration of 200 ng/mL to mouse primary astrocytes for 2 h. The results revealed that recombinant mouse CK2 stimulation significantly induced the expression of C3, CXCL10, and STEAP4 in mouse primary astrocytes (Fig 3E and 3F). The role of CK2 in the meningitic *E. coli* infection-induced astrocyte reaction was further analyzed in a mouse model. The mice were administered CX4945 (2.5 mg/kg), an inhibitor of CK2 activity, via intravenous injection 2 h before infection (Fig 3G). At 2 hpi, the brain hippocampal tissues of the mice were harvested to analyze the expression of astrocyte pan-activation markers, LPS-specific reactive markers, and pro-inflammatory cytokines via real-time PCR. As shown in Fig 3H, the meningitic *E. coli* challenge resulted in significant upregulation of astrocyte pan-activation markers (*Gfap*, *Steap4* and *Cxcl10*), LPS-specific reactive markers (*C3*, *Iigp1* and *Amigo2*), and pro-inflammatory cytokines (*TNF-α*, *IL-1β* and *IL-6*). The above *in vivo* and *in vitro* data collectively indicate that CK2 plays a significant role in the induction of the astrocyte reaction.

### CK2 induces astrocyte reaction by activating the NF-κB signaling pathway

We subsequently sought to elucidate the molecular mechanisms by which CK2 induces the astrocyte reaction, thereby promoting neuroinflammation. We selected several classical signaling pathways with established roles in regulating astrocyte reaction and *Gfap* transcription, including the JAK/STAT, PI3K-Akt, ERK1/2, JNK, and NF-κB pathways, for testing. Mouse primary astrocytes were pretreated with 10 μM of the JAK/STAT-specific inhibitor Ruxolitinib, 10 μM of the PI3K-Akt-specific inhibitor Wortmannin, 10 μM of the ERK1/2-specific inhibitor U0126, 10 μM of the JNK-specific inhibitor SP600125 and 10 μM of the NF-κB-specific inhibitor BAY 11–7082, and the results demonstrated that the transcriptional upregulation of *Gfap* induced by CK2 was significantly suppressed by BAY 11–7082, Ruxolitinib, Wortmannin and SP600125. Among them, the level of *Gfap* in the BAY 11–7082 treatment group decreased the most significantly. (Fig 4A). The activation of the NF-κB pathway was subsequently investigated following the treatment of mouse primary astrocytes with mouse recombinant CK2. The results of the western blot analysis demonstrated that CK2 stimulation dose-dependently and time-dependently promoted the phosphorylation of p65, which led to the activation of the NF-κB signaling pathway (Fig 4B–4E). Subsequent investigations demonstrated that the inhibition of the NF-κB signaling pathway led to a notable reduction in the inflammatory response induced by mCK2 in mouse primary astrocytes. As illustrated in Fig 4F, the mCK2-induced upregulation of astrocyte pan-activation markers (*Gfap*, *Cxcl10* and *Steap4*), LPS-specific reactive markers (*C3*, *Iigp1* and *Amigo2*), and pro-inflammatory cytokines (*Tnf-α* and *Il-6*) in mouse primary astrocytes were markedly suppressed by the NF-κB-specific inhibitor BAY 11–7082. And the CK2-induced pro-inflammatory cytokines upregulation in U251 (*TNF-α*, *IL-6, CXCL3* and *CXCL10*) were also blocked by BAY 11–7082 treatment (Fig 4G). The role of the NF-κB signaling pathway in CK2-induced neuroinflammation was further analyzed in a mouse model (Fig 4H). Mice were administered BAY 11–7082 (1.5 mg/kg) and a total of 0.3 μg/kg of mCK2 via intravenous injection for 2 h, and the brain hippocampal tissues from the mice were harvested to perform the real-time PCR analysis, and the results were in accordance with those of the *in vitro* analysis (Fig 4I). The inhibition of the NF-κB signaling pathway resulted in a significant reduction of astrocyte pan-activation markers (*Gfap*, *Cxcl10* and *Steap4*), LPS-specific reactive markers (*C3*, *Iigp1* and *Amigo2*), and pro-inflammatory cytokines (*Tnf-α*, *Il-1β* and *Il-6*) transcription induced by CK2. The *in vitro* and *in vivo* findings indicate that CK2 induces neuroinflammation by activating NF-κB signaling.

### Myosin 9 mediates the CK2-induced astrocyte reaction

To identify the endogenous proteins that interact with extracellular CK2, U251 cells were incubated with 1 μg/mL recombinant CK2 (His-tagged CK2α and GST-tagged CK2β) for 3 h, after which the cell lysate supernatants were immunoprecipitated with mouse IgG, mouse-derived His-tag antibody, and mouse-derived GST tag antibody, respectively. By SDS-PAGE we detected a distinct protein band at approximately 180 kDa in the upstream lane of the GST-tag antibody incubated lysate from the CK2 protein-treated cells compared with the CK2 reconstitution buffer-treated cells. We next employed LC-MS/MS to analyze the protein composition of this specific band. By further analysis of the average peptide coverage and number of unique peptides, we identified myosin 9 as a target protein for CK2 (Fig 5A, S4 Table). Immunoblotting was used to analyze the co-precipitation of endogenous myosin 9 and CK2. CDT, which has been reported as a cell-binding exotoxin, was used as a His-/GST-labeled recombinant protein control [34]. As shown in Fig 5B, we found that endogenous myosin 9 significantly interacts with the CK2β subunit of CK2. The co-localization of extracellular CK2 and myosin 9 was also confirmed by immunofluorescence experiments, in which the recruitment of myosin 9 was observed in CK2-treated cells. We found that CK2β rather than CK2α co-localized with myosin 9, which was consistent with the Co-IP results (Fig 5C). The above results implied that exogenous CK2β can recruit and bind to myosin 9 in astrocytes. Next, the truncated forms of *CSNK2B* were cloned and inserted into pEGFP-C1 with an N-terminal GFP tag based on the reported domain architecture of CK2β [35] (S4A Fig). The truncated constructs of CK2β were then overexpressed in HEK293T cells, and the cells were lysed and subjected to an immunoprecipitation assay with an anti-myosin 9 antibody. The results revealed that CK2β_91–215_ interacted with myosin 9 (Fig 5D). Overall, we have shown that the β-subunit of CK2 recruits myosin 9 and interacts with myosin 9 through its amino acids 91–215.

Based on the evidence that exogenous CK2 interacts with myosin 9, we investigated the potential involvement of the CK2-myosin 9 interaction in regulating the inflammatory response of astrocytes. Western blot analysis demonstrated that treatment of astrocytes with 100 ng/mL CK2 resulted in a significant time-dependent increase in the phosphorylation of myosin 9 and p65 of the mouse primary astrocytes. Furthermore, our observations indicated that CK2-induced phosphorylation of myosin 9 preceded that of p65 (S4B–S4C Fig). In contrast, treatment of 100 ng/mL α or β subunit alone was ineffective at inducing myosin 9 and p65 phosphorylation, respectively (Figs 5E and S4D). To investigate the regulatory relationship between myosin 9 and p65, we employed the CRISPR-Cas9 technique to delete myosin 9 in U251 cells. As shown in S5A Fig, myosin 9 was effectively knocked out in U251 cells. Subsequently, wild-type and myosin 9-KO U251 cells were treated with CK2, and the results of a western blot analysis indicated that myosin 9 deletion resulted in partial inhibition of p65 phosphorylation (Figs 5F and S4E). The real-time PCR results also revealed that U251 cells lacking myosin 9 presented significantly lower some of the CK2-induced pro-inflammatory factors, including *TNF-α*, *IL-1β* and *CXCL10* (Fig 5H). Moreover, our findings indicate that CK2-induced phosphorylation of myosin 9 remains unaltered by pretreatment with the NF-κB-specific inhibitor BAY 11–7082 (Figs 5G and S4F). Additionally, myosin 9-KO U251 cells were utilized in the coculture model, and real-time PCR results indicated that myosin 9 depletion also reduced the transcription of meningitic *E. coli*-induced pro-inflammatory factors (Fig 5I and 5J). These results demonstrate that CK2 promotes the astrocyte response by regulating myosin 9 translocation and triggering downstream NF-κB activation.

### CK2 deficiency in BMEC attenuates neuroinflammation induced by meningitic *E. coli*

It has been demonstrated that the complete absence of both CK2α and CK2β in mice is embryonically lethal because of the essential role of CK2 in development. Even conditional KO mice targeting CK2β in the central nervous system died shortly after birth [36,37]. Considering the potential impact of the loss of the catalytic subunit α in BMEC on the cell cycle, we investigated the effect of BMEC-derived CK2 on the astrocyte reaction via the knockdown of the β subunit, which is essential for the export of the CK2 holoenzyme [38]. As shown in S5B Fig, the CK2β protein was effectively knocked out in hBMEC. To ascertain whether CK2β plays a role in regulating CK2 extracellular export, the supernatants and lysates of wild-type and CK2β-knockdown hBMEC cell culture were collected. Western blot analysis revealed the absence of extracellular CK2α in the supernatant of CK2β-deficient hBMEC, even in the presence of meningitic *E. coli* infection (Fig 6A). Additionally, CK2β-deficient hBMEC were utilized in the coculture model, and real-time PCR results indicated that CK2β depletion also reduced some of the meningitic *E. coli*-induced pro-inflammatory factors transcription, including *TNF-α*, *IL-8*, *MIP2*, *CXCL3* and *CXCL10* (Fig 6B and 6C). To further elucidate the function of endothelial CK2β in astrocyte reaction in response to meningitic *E. coli*, a vascular-specific knockout (*CK2βVas*^*-/-*^) mouse model was generated through Cre/LoxP-mediated genome engineering and knockout identification was validated by PCR (S5C Fig). *CK2βVas*^*-/-*^ and wild-type mice were treated with tamoxifen for 7 days. Subsequently, isolated cerebrovascular and whole-brain tissues were examined by western blot for CK2β expression. The results demonstrated that CK2β expression was undetectable in cerebrovascular samples derived from *CK2βVas*^*-/-*^ mice, whereas CK2β expression was detected in the *CK2βVas*^*-/-*^ mice whole-brain tissue. In contrast, CK2β expression was detected in both cerebrovascular samples and whole-brain tissue from wild-type mice (S5D Fig). Moreover, immunofluorescence was used to determine the expression of CK2β in the cerebral vessels of *CK2βVas*^*-/-*^ mice. The cerebrovascular was labeled with CD31, and no evidence of CK2β expression was detected in the cerebrovascular of *CK2βVas*^*-/-*^ mice (S5E Fig). The above results demonstrate the successful generation of mice with conditional deficiency for CK2β in vascular tissue. To further determine the effects of CK2β on astrocyte reaction and neuroinflammation induced by meningitic *E. coli*, immunofluorescence was performed to examine the expression of CXCL10, STEAP4, C3 and GFAP in infected wild-type and *CK2βVas*^*-/-*^ mice. It was observed that meningitic *E. coli* infection of wild-type mice resulted in a notable increase in GFAP fluorescence intensity. The expression of CXCL10, STEAP4, and C3 were also significantly upregulated in brain tissue. Conversely, infection-induced upregulation of CXCL10, STEAP4, C3 and GFAP was significantly reduced in *CK2βVas-/-* mice (Figs 6D–6F, S5F–S5H). Furthermore, we subsequently assessed its ability to regulate astrocyte pan-activation markers, LPS-specific reactive markers, and pro-inflammatory cytokines transcription in response to *in vivo* infection via real-time PCR. We found that *Gfap*, *Cxcl10*, *Steap4*, *C3*, *Iigp1*, *Amigo2*, *TNF-α*, *IL-1β* and *IL-6* levels in the hippocampus were significantly augmented in bacterium-infected wild-type mice; however, the upregulation of these inflammatory molecules was significantly reduced in bacterium-infected *CK2βVas*^*-/-*^ mice (Fig 6G). Therefore, our results revealed that loss of CK2β in vascular endothelial cells significantly affects astrocyte reaction in the early stage of *E. coli* meningitis. Considering the deleterious effect of CK2β on the exacerbation of neuroinflammation in mice infected with meningitic *E. coli*, we investigated whether CK2β knockout could confer protection against meningitic *E. coli*-induced mortality in mice. As evidenced by the results, all mice in the uninfected wild-type and *CK2βVas*^*-/-*^ groups survived throughout the observation period. In contrast, the mortality rate among wild-type mice infected with bacteria was 100%. Nevertheless, CK2β knockout afforded protection from mortality in 42% of the *CK2βVas*^*-/-*^ mice with bacterial infection (Fig 6H). These results indicate that CK2β is indispensable for the transportation and extracellular localization of CK2. Deprivation of CK2β in BMEC prevents meningitic *E. coli*-induced astrocyte reaction and neuroinflammation, thereby affording the host a certain degree of protection.

## Discussion

As key cellular components of the central nervous system, astrocytes play a supportive role under physiological conditions and are involved in regulating various processes, such as synapse development, ion metabolism, and extracellular space formation [39]. In pathological conditions including neurodegenerative diseases, infections, and trauma, astrocytes also act as pivotal regulators of neuroinflammation [40–42]. The upregulation of GFAP serves as an established marker for astrocyte reaction. Previous research has focused predominantly on GFAP upregulation in reactive astrogliosis under chronic pathological conditions. On the other hand, GFAP can also elicit an immediate response to certain stimuli. For example, an increase in the GFAP signal was observed as early as 0.5 h in cryogenic lesions of the rat brain [43]. In a mouse model of blood-borne *Streptococcus pneumoniae* infection, the upregulation of GFAP expression was noted as early as 1 hpi [44]. Here, our results provide important evidence indicating that meningitic *E. coli* contributes to the astrocyte reaction 2 hpi, as indicated by the upregulation of GFAP and the increase of S100β and GFAP double-positive cells within the hippocampal region. Interestingly, as another region with high GFAP immunostaining, the fluorescence intensity of GFAP in the internal capsule in 2 hpi group was not significantly different from that of the control group, and the temporal differences in the astrocyte reaction states in these two regions are worthy of further exploration. While virulence factors or components of pathogens can induce astrocyte reaction by binding to TLRs, negative results from the extravasation of Evans blue and albumin indicated maintained vascular integrity, similar to what Iovino and colleagues observed in their research, suggesting that the astrocyte reaction was not directly caused by pathogenic components crossing the blood-brain barrier and entering the brain parenchyma in the early phase of meningitic *E. coli* infection [44,45]. Studies have shown that communication with other cell types in the central nervous system can induce astrocyte reaction. Activated microglia have been shown to induce reactive astrogliosis both in mouse models with LPS treatment and in mouse models exhibiting depressive-like behavior; however, no signs of microglial activation were observed in the early stages of infection in our study according to the morphological analysis of Iba-1 positive cells in the hippocampal region [33,46]. Vascular endothelial cells are also capable of driving astrocyte reactivity, which is different from that induced by microglia [14]. Moreover, endothelial cells play crucial roles in inducing inflammatory responses associated with reactive astrogliosis within cerebral cavernous malformations [13]. We demonstrated *in vitro* that astrocytes in a coculture system could be activated by endothelial cells infected with meningitic *E. coli*, as indicated by the upregulation of reactive markers and pro-inflammatory cytokines. However, unlike the 2 hpi group, the level of microglia activation and the degree of vascular leakage in the brains of mice in the 12 hpi group were significantly increased. Meanwhile, we also observed the exudation of polymorphonuclear neutrophils (Ly6G positive cells) in the brain of 12 hpi, suggesting that the significant astrocyte reaction at this time might be the result of the synergistic effect of these multiple factors (S1J–S1K Fig).

Previous systematic studies on astrocyte reaction revealed that under various pathological conditions, distinct markers may exhibit specifically elevated expression levels in astrocytes. Some studies have classified astrocytes into two subtypes, A1 and A2, corresponding to neurotoxic and neurosupportive properties, respectively, through transcriptomic analysis [11]. As the research progressed, it was discovered that this definition method had obvious limitations and could not serve as a universal standard to generalize all scenarios. Therefore, at present, the determination of reactive activation of astrocytes still requires, on the basis of combining actual disease models and cell morphological analysis, the detection of the expression levels of potential markers (such as GFAP, Vimentin, C3, S100β, SOX9 and KIR 4.1, etc.). Although this nomenclature has been abandoned, we noticed that the significant upregulation of some genes in LPS-treated mice (PAN-reactive and LPS-specific) were also verified in the activated astrocytes in this study, suggesting that LPS may play an important role in astrocyte reaction induced by *E. coli* meningitis. Numerous studies have shown that neurotoxic astrocytes induced by PAMPs and DAMPs play a role as boosters in inflammatory injury to the central nervous system [47,48]. In *Toxoplasma gondii* infection, compared with the control and chronic infection groups, acute infection or treatment with Toxoplasma antigens can induce reactive astrocytes and high expression of C3, which eventually leads to neuronal apoptosis [49]. In mouse models of EAE, the NLRP3 inflammasome from microglia induces astrocyte reaction, leading to synaptic loss and nerve damage in the hippocampus [50]. In this study, we found that CK2, which is upregulated in the secreted proteome of BMEC after meningitic *E. coli* stimulation, may have the potential to induce astrocyte reaction and inflammation. Therefore, in addition to microglia, communication with other resident cells in the brain (such as BMEC) may also be an important factor in inducing the activation of astrocytes.

This study for the first time identified CK2 as an extracellular kinase that can affect intracellular processes and provided an in-depth understanding of the specific mechanism involved. CK2 is best known for its role in cancer therapy because of its involvement in regulating cell survival and proliferation pathways. Nevertheless, its role as an extracellular kinase has been poorly studied [51–53]. Ectokinases are generally considered to be the extracellular version of the intracellular kinase. To date, at least 15 proteins have been identified as substrates of extracellular CK2 (ecto-CK2), which are involved in various extracellular processes such as coagulation and cell adhesion [19,54]. In the present study, recombinant CK2 induced astrocyte reaction *in vitro* and *in vivo*. It has been found that pathways such as JAK/STAT3, NF-κB, CN and MAPK may be associated with the reaction of astrocytes [55–58]. NF-kB is one of the most widely reported major pathways regulated by CK2 [59]. Among the inhibitors targeting the pathways mentioned above, BAY 11–7082 has the strongest inhibitory effect on CK2-induced astrocyte reaction and inflammation. These results highlight the importance of the NF-kB pathway in the regulation of astrocyte reaction induced by extracellular CK2. In addition, pre-treatment of Ruxolitinib, SP600125, Wortmannin also exhibited significant inhibition of CK2 function, suggesting that multiple signaling pathways may be involved in astrocyte reaction induced by CK2 based on its broad-spectrum kinase activity.

Myosin is widely known as a molecular motor. Unlike muscle myosin which assists in muscle contraction, non-muscle myosin is distributed in all eukaryotic cells, where it participates in a variety of cellular processes that require the movement of the actin skeleton [60,61]. It has been shown that myosin 9, the heavy chain of non-muscle myosin of class II, isoform A (NMIIA), is a substrate of CK2. The phosphorylation of myosin 9 affects the assembly of NMII and the binding of associated proteins, but the mechanism of interaction between CK2 and myosin 9 is still unclear [62]. As a regulatory subunit, CK2β is more responsible for substrate anchoring than the catalytic subunit CK2α is [63]. The interaction between CK2β and myosin 9 was confirmed by immunoprecipitation and indirect immunofluorescence. Surprisingly, we found the presence of exogenous CK2β in the astrocyte lysates after the CK2 treatment, but no CK2α was detected in either of these results. This phenomenon indicates that CK2β may attach to astrocytes by binding to myosin 9, while CK2α somehow dissociates from β-subunit. Besides, further verification is required to explore whether CK2β can penetrate the cytomembrane and enter into the cytoplasm. However, in contrast to treatment with holoenzyme, treatment with neither the α-subunit nor the β-subunit alone induced the phosphorylation of myosin 9 and the activation of NF-κB in astrocytes, indicating that both the α and β subunits are required for the function of CK2. In addition, we found that myosin 9 is associated with CK2-induced NF-κB pathway activation. Deletion of myosin 9 inhibited p65 phosphorylation mediated by CK2. The myosin 9-p53/Rho-p38 pathway was previously found to regulate NF-kB activation in gastric cancer cells, indicating a relationship between myosin 9 and NF-kB [64]. However, how CK2 regulates NF-kB activation through myosin 9 needs further investigation.

There are still some limitations in this study. First, during the development of bacterial meningitis, transcellular transport is also one of the main ways for *E. coli* to cross the BBB. In the co-culture model of BMEC and astrocytes, we examined the ability of bacteria to penetrate BMEC monolayer. Supernatant of astrocyte cultures from each biological repetition were collected after infection and all of them were inoculated on LB plates for bacterial cultivation, and almost no live bacteria could be detected in the cell culture supernatant. However, we are unable to determine whether the bacterial products can enter into the lower chamber. Also, the changes of transcellular transport will unlikely be detected through Evans blue assays *in vivo*, and more comprehensive detection methods should be employed to assess the permeability of BBB. Second, In this study, human astrocytoma cell line U251 instead of mouse primary astrocytes was used in some of the experiments (immunofluorescence and generation of the KO cell lines) because of the excessively long growth cycle and difficulty in being transfected of primary cells. Even though some of the conclusions obtained from the U251 cells may not conflict with the experimental results *in vivo*, we will still seek more appropriate solutions or find more suitable cell lines to supplement and prove the existing conclusions. Third, some of the activation indicators of astrocytes that we have detected (especially the inflammatory response-related cytokines) are not unique to astrocytes, other cell types in the brain, such as microglia cells and neurons, are also important sources. Whether secreted CK2 during bacterial infection acts on these cells to produce the same effect, and how to conduct more specific detection of astrocyte reaction are the goals we need to achieve in the future.

## Conclusions

In summary, we elucidated the mechanism by which secreted protein-mediated endothelial-glial communication promotes astrocyte reaction and the inflammatory response during meningitic *E. coli* infection. We also demonstrated that CK2 interacted with myosin 9 to activate the downstream NF-kB pathway, providing new insights into the function of CK2 as an extracellular kinase. This study provides new ideas and directions for the research of molecular targets and treatment methods for central nervous system inflammation induced by bacterial blood-borne infection.

## Supporting information

S1 Fig Astrocyte reactivity at the early stage of meningitic *E. coli* infection. (A) Immunofluorescence of GFAP (green) in control, 2 hpi and 12 hpi mouse brains. 1, hippocampal region, 2, internal capsule region. Scale bar, 1000 μm and 100 μm. Quantification of GFAP fluorescence intensity in the (B) hippocampal region and (C) internal capsule region. n = 3 mice per group. (D) Immunofluorescence of S100β (red) in control, 2 hpi and 12 hpi mouse brains. 1, hippocampal region, 2, internal capsule region. Scale bar, 1000 μm and 100 μm. Quantification of S100β fluorescence intensity in the (E) hippocampal region and (F) internal capsule region. n = 3 mice per group. (G) Four fixed regions within the hippocampal region used for microglia statistical analysis of Figs 1B, 1E-1G and S1I. Scale bar, 2000 μm. (H) Immunofluorescence of CD31 and Albumin in the mouse brain of control, 2 hpi and 12 hpi group. Scale bar, 2 mm and 50 μm. (I) Pearson’s correlation coefficients of albumin and CD31 in control, 2 hpi and 12 hpi mouse brains (n = 3 mice per group. For each mouse, four fixed regions per section were analyzed. The median value from these four regions in all three mice was used for statistical analysis). (J) Immunofluorescence of Ly6G (red) in 12 hpi mouse brain. 1, meningeal region, 2, hippocampal region, 3, subventricular zone. White arrows indicate Ly6G positive cells. Scale bar, 1000 μm. (K) Number of Ly6G positive cells in control, 2 hpi and 12 hpi mouse brains. n = 3 mice per group. (L) hBMEC in upper chamber was challenged for 2 h with 10 MOI meningitic *E. coli* or left untreated; mRNA fold changes of pro-inflammatory factors in U251 after infection. n = 3. Data are shown as mean ± SEM. One-way ANOVA (B, C, E, F, I, K) followed by Tukey’s multiple comparison and Two-way ANOVA (L) followed by Bonferroni’s multiple comparison test; *p < 0.033, ** p < 0.002, *** p < 0.001, ns, not significant.
(TIF)

S2 Fig Quantitative analysis of the DEPs expression in the cell culture supernatant of BMEC in response to meningitic *E. coli* infection. Analysis results are presented as DEPs/loading control (ACTN4). n = 3. Data are shown as mean ± SEM.
(TIF)

S3 Fig Enzyme activity and Cytotoxicity of CK2 subunits and holoenzyme. (A) Enzyme activity of CK2 subunits and holoenzyme was reflected by ATP consumption after the incubation of 50 ng CK2α, mCK2α, CK2 and mCK2 with equivalent U251 cell lysates for 10 min at 37 °C. n = 3. Data are shown as mean ± SEM. One-way ANOVA followed by Tukey’s multiple comparison. **p < 0.002, ***p < 0.001. (B) Cytotoxicity of CK2 subunits and holoenzyme was reflected by cell viability measured with a Cell Counting Kit-8. Astrocytes in 96-well plates were treated by 400 ng/mL mCK2α, CK2α, mCK2β, CK2β, mCK2 and CK2 for 1 h, five parallel wells were used for each treatment.
(TIF)

S4 Fig CK2 induces NF-κB activation through myosin9. (A) Illustration of CK2β truncated mutant constructs with amino-terminal GFP fusions. (B) Western blot of astrocytic p65/myosin 9 phosphorylation induced by CK2 in a time gradient in mouse primary astrocytes. n = 3. (C) Fold changes of phosphorylated myosin 9/myosin 9 and phosphorylated p65/p65 in mouse primary astrocytes after CK2 treatment. n = 3. (D) Fold changes of phosphorylated myosin 9, myosin 9, phosphorylated p65 and p65 in mouse primary astrocytes at 30 min after 100 ng/mL CK2α, CK2β and CK2 treatment. n = 3. (E) Fold changes of myosin 9, phosphorylated p65 and p65 in WT and myosin 9-KO U251 cells after 100 ng/mL CK2 treatment for 1 h. n = 3. (F) Fold changes of phosphorylated myosin 9 and myosin 9 in mouse primary astrocytes pretreated with BAY 11–7082 (NF-κB inhibitor) or vehicle (DMSO) following 100 ng/mL CK2 treatment for 10 min. n = 3. Data are shown as mean ± SEM. Two-way ANOVA (C, D, E, F) followed by Bonferroni’s multiple comparison tests; *p < 0.033, ** p < 0.002, *** p < 0.001, ns, not significant.
(TIF)

S5 Fig Detection of knockdown efficiency of myosin9 and CK2β in cultured cells and identification of CK2β conditional knockout mice in mock/infected conditions. (A)Western blot results of myosin 9-KO U251 cell line screening. n = 3. (B) Western blot results of CK2β-KO hBMEC cell line screening. n = 3. (C) PCR-based genotyping screening for CK2β conditional knockout mice, which detected the DNA bands at 199 bp for homozygotes with loxP sites in *CSNK2B* gene and 248 bp for Cre recombinase (for example, #11). (D) CK2β expression in cerebral microvessel samples and brain tissue samples from WT and *CK2βVas*^*-/-*^ mice. n = 3. (E) Immunostaining of CK2β (green) and the vascular endothelial marker CD31 (red) in the brain parenchyma of WT and *CK2βVas*^*-/-*^mice 2 h after infection. Scale bar, 50 μm. Quantification of (F) STEAP4, (G) CXCL10, and (H) C3 (all in red) with GFAP (green) in the hippocampus of WT and *CK2βVas*^*-/-*^ mice post 2 h infection. Scale bar, 20 μm. n = 3. Data are shown as mean ± SEM. Two-way ANOVA (F, G, H) followed by Bonferroni’s multiple comparison test. *p < 0.033, ** p < 0.002, *** p < 0.001, ns, not significant.
(TIF)

S1 Table Primers, sgRNA and siRNA used in the study. (DOCX)

S2 Table Secreted proteins of DEPs. (XLSX)

S3 Table KEGG analysis of secreted proteins in DEPs (top 10 pathways). (XLSX)

S4 Table LC-MS/MS analysis of the protein composition in the 200kDa SDS-PAGE band from the samples that CK2 immunoprecipitated with U251 cell lysates. (XLSX)

S5 Table Antibodies used in the study. (DOCX)

S1 Data Raw data of SDS-PAGE and western blot in this study. (DOCX)

S2 Data Collection of fluorescence images (Figs 1–6). (PDF)

## References

[1] Edmond K, Clark A, Korczak VS, Sanderson C, Griffiths UK, Rudan I. Global and regional risk of disabling sequelae from bacterial meningitis: a systematic review and meta-analysis. Lancet Infect Dis. 2010;10(5):317–28. doi: 10.1016/S1473-3099(10)70048-7 20417414

[2] Yang R, Wang J, Wang F, Zhang H, Tan C, Chen H, et al. Blood-Brain Barrier Integrity Damage in Bacterial Meningitis: The Underlying Link, Mechanisms, and Therapeutic Targets. Int J Mol Sci. 2023;24(3):2852. doi: 10.3390/ijms24032852 36769171 PMC9918147

[3] Kim KS. Acute bacterial meningitis in infants and children. Lancet Infect Dis. 2010;10(1):32–42. doi: 10.1016/S1473-3099(09)70306-8 20129147

[4] Kim KS, Itabashi H, Gemski P, Sadoff J, Warren RL, Cross AS. The K1 capsule is the critical determinant in the development of Escherichia coli meningitis in the rat. J Clin Invest. 1992;90(3):897–905. doi: 10.1172/JCI115965 1326000 PMC329944

[5] Kim KS. Human Meningitis-Associated Escherichia coli. EcoSal Plus. 2016;7(1):10.1128/ecosalplus.ESP-0015–2015. doi: 10.1128/ecosalplus.ESP-0015-2015 27223820 PMC4881430

[6] Han RT, Kim RD, Molofsky AV, Liddelow SA. Astrocyte-immune cell interactions in physiology and pathology. Immunity. 2021;54(2):211–24. doi: 10.1016/j.immuni.2021.01.013 33567261

[7] Yang R, Yang B, Liu W, Tan C, Chen H, Wang X. Emerging role of non-coding RNAs in neuroinflammation mediated by microglia and astrocytes. J Neuroinflammation. 2023;20(1):173. doi: 10.1186/s12974-023-02856-0 37481642 PMC10363317

[8] Escartin C, Guillemaud O, Carrillo-de Sauvage M-A. Questions and (some) answers on reactive astrocytes. Glia. 2019;67(12):2221–47. doi: 10.1002/glia.23687 31429127

[9] Grimaldi A, Pediconi N, Oieni F, Pizzarelli R, Rosito M, Giubettini M, et al. Neuroinflammatory Processes, A1 Astrocyte Activation and Protein Aggregation in the Retina of Alzheimer’s Disease Patients, Possible Biomarkers for Early Diagnosis. Front Neurosci. 2019;13:925. doi: 10.3389/fnins.2019.00925 31551688 PMC6737046

[10] Yun SP, Kam T-I, Panicker N, Kim S, Oh Y, Park J-S, et al. Block of A1 astrocyte conversion by microglia is neuroprotective in models of Parkinson’s disease. Nat Med. 2018;24(7):931–8. doi: 10.1038/s41591-018-0051-5 29892066 PMC6039259

[11] Liddelow SA, Guttenplan KA, Clarke LE, Bennett FC, Bohlen CJ, Schirmer L, et al. Neurotoxic reactive astrocytes are induced by activated microglia. Nature. 2017;541(7638):481–7. doi: 10.1038/nature21029 28099414 PMC5404890

[12] Li W, Mandeville ET, Durán-Laforet V, Fukuda N, Yu Z, Zheng Y, et al. Endothelial cells regulate astrocyte to neural progenitor cell trans-differentiation in a mouse model of stroke. Nat Commun. 2022;13(1):7812. doi: 10.1038/s41467-022-35498-6 36535938 PMC9763251

[13] Lai CC, Nelsen B, Frias-Anaya E, Gallego-Gutierrez H, Orecchioni M, Herrera V, et al. Neuroinflammation Plays a Critical Role in Cerebral Cavernous Malformation Disease. Circ Res. 2022;131(11):909–25. doi: 10.1161/CIRCRESAHA.122.321129 36285625 PMC9669201

[14] Taylor X, Cisternas P, Jury N, Martinez P, Huang X, You Y, et al. Activated endothelial cells induce a distinct type of astrocytic reactivity. Commun Biol. 2022;5(1):282. doi: 10.1038/s42003-022-03237-8 35351973 PMC8964703

[15] Meggio F, Pinna LA. One-thousand-and-one substrates of protein kinase CK2? FASEB J. 2003;17(3):349–68. doi: 10.1096/fj.02-0473rev 12631575

[16] Singh NN, Ramji DP. Protein kinase CK2, an important regulator of the inflammatory response? J Mol Med (Berl). 2008;86(8):887–97. doi: 10.1007/s00109-008-0352-0 18437331

[17] Buontempo F, McCubrey JA, Orsini E, Ruzzene M, Cappellini A, Lonetti A, et al. Therapeutic targeting of CK2 in acute and chronic leukemias. Leukemia. 2018;32(1):1–10. doi: 10.1038/leu.2017.301 28951560 PMC5770594

[18] Yamaguchi Y, Wada T, Suzuki F, Takagi T, Hasegawa J, Handa H. Casein kinase II interacts with the bZIP domains of several transcription factors. Nucleic Acids Res. 1998;26(16):3854–61. doi: 10.1093/nar/26.16.3854 9685505 PMC147779

[19] Montenarh M, Götz C. Ecto-protein kinase CK2, the neglected form of CK2. Biomed Rep. 2018;8(4):307–13. doi: 10.3892/br.2018.1069 29556379 PMC5844033

[20] Liu C, Zheng H, Yang M, Xu Z, Wang X, Wei L, et al. Genome analysis and in vivo virulence of porcine extraintestinal pathogenic Escherichia coli strain PCN033. BMC Genomics. 2015;16(1):717. doi: 10.1186/s12864-015-1890-9 26391348 PMC4578781

[21] Takagaki Y, Lee SM, Dongqing Z, Kitada M, Kanasaki K, Koya D. Endothelial autophagy deficiency induces IL6 - dependent endothelial mesenchymal transition and organ fibrosis. Autophagy. 2020;16(10):1905–14. doi: 10.1080/15548627.2020.1713641 31965901 PMC8386622

[22] Yang R, Chen J, Qu X, Liu H, Wang X, Tan C, et al. Interleukin-22 Contributes to Blood-Brain Barrier Disruption via STAT3/VEGFA Activation in Escherichia coli Meningitis. ACS Infect Dis. 2024;10(3):988–99. doi: 10.1021/acsinfecdis.3c00668 38317607 PMC10928716

[23] Yang R, Qu X, Zhi S, Wang J, Fu J, Tan C, et al. Exosomes Derived from Meningitic Escherichia coli-Infected Brain Microvascular Endothelial Cells Facilitate Astrocyte Activation. Mol Neurobiol. 2024;61(9):7195–210. doi: 10.1007/s12035-024-04044-4 38372957

[24] Guo Q, Gobbo D, Zhao N, Zhang H, Awuku N-O, Liu Q, et al. Adenosine triggers early astrocyte reactivity that provokes microglial responses and drives the pathogenesis of sepsis-associated encephalopathy in mice. Nat Commun. 2024;15(1):6340. doi: 10.1038/s41467-024-50466-y 39068155 PMC11283516

[25] Ahlemeyer B, Kehr K, Richter E, Hirz M, Baumgart-Vogt E, Herden C. Phenotype, differentiation, and function differ in rat and mouse neocortical astrocytes cultured under the same conditions. J Neurosci Methods. 2013;212(1):156–64. doi: 10.1016/j.jneumeth.2012.09.016 23026192

[26] Bernard-Patrzynski F, Lécuyer M-A, Puscas I, Boukhatem I, Charabati M, Bourbonnière L, et al. Isolation of endothelial cells, pericytes and astrocytes from mouse brain. PLoS One. 2019;14(12):e0226302. doi: 10.1371/journal.pone.0226302 31851695 PMC6919623

[27] Fu J, Li L, Huo D, Zhi S, Yang R, Yang B, et al. Astrocyte-Derived TGFβ1 Facilitates Blood-Brain Barrier Function via Non-Canonical Hedgehog Signaling in Brain Microvascular Endothelial Cells. Brain Sci. 2021;11(1):77. doi: 10.3390/brainsci11010077 33430164 PMC7826596

[28] Young K, Morrison H. Quantifying Microglia Morphology from Photomicrographs of Immunohistochemistry Prepared Tissue Using ImageJ. J Vis Exp. 2018;(136):57648. doi: 10.3791/57648 29939190 PMC6103256

[29] Pancetti F, Bosser R, Krehan A, Pyerin W, Itarte E, Bachs O. Heterogeneous nuclear ribonucleoprotein A2 interacts with protein kinase CK2. Biochem Biophys Res Commun. 1999;260(1):17–22. doi: 10.1006/bbrc.1999.0849 10381337

[30] Lawrence JM, Schardien K, Wigdahl B, Nonnemacher MR. Roles of neuropathology-associated reactive astrocytes: a systematic review. Acta Neuropathol Commun. 2023;11(1):42. doi: 10.1186/s40478-023-01526-9 36915214 PMC10009953

[31] Kabba JA, Xu Y, Christian H, Ruan W, Chenai K, Xiang Y, et al. Microglia: Housekeeper of the Central Nervous System. Cell Mol Neurobiol. 2018;38(1):53–71. doi: 10.1007/s10571-017-0504-2 28534246 PMC11481884

[32] Rothhammer V, Borucki DM, Tjon EC, Takenaka MC, Chao C-C, Ardura-Fabregat A, et al. Microglial control of astrocytes in response to microbial metabolites. Nature. 2018;557(7707):724–8. doi: 10.1038/s41586-018-0119-x 29769726 PMC6422159

[33] Zamanian JL, Xu L, Foo LC, Nouri N, Zhou L, Giffard RG, et al. Genomic analysis of reactive astrogliosis. J Neurosci. 2012;32(18):6391–410. doi: 10.1523/JNEUROSCI.6221-11.2012 22553043 PMC3480225

[34] Zhou M, Zhang Q, Zhao J, Jin M. Haemophilus parasuis encodes two functional cytolethal distending toxins: CdtC contains an atypical cholesterol recognition/interaction region. PLoS One. 2012;7(3):e32580. doi: 10.1371/journal.pone.0032580 22412890 PMC3296717

[35] Bolanos-Garcia VM, Fernandez-Recio J, Allende JE, Blundell TL. Identifying interaction motifs in CK2beta--a ubiquitous kinase regulatory subunit. Trends Biochem Sci. 2006;31(12):654–61. doi: 10.1016/j.tibs.2006.10.005 17084631

[36] Huillard E, Ziercher L, Blond O, Wong M, Deloulme J-C, Souchelnytskyi S, et al. Disruption of CK2beta in embryonic neural stem cells compromises proliferation and oligodendrogenesis in the mouse telencephalon. Mol Cell Biol. 2010;30(11):2737–49. doi: 10.1128/MCB.01566-09 20368359 PMC2876519

[37] Lou DY, Dominguez I, Toselli P, Landesman-Bollag E, O’Brien C, Seldin DC. The alpha catalytic subunit of protein kinase CK2 is required for mouse embryonic development. Mol Cell Biol. 2008;28(1):131–9. doi: 10.1128/MCB.01119-07 17954558 PMC2223292

[38] Rodríguez F, Allende CC, Allende JE. Protein kinase casein kinase 2 holoenzyme produced ectopically in human cells can be exported to the external side of the cellular membrane. Proc Natl Acad Sci U S A. 2005;102(13):4718–23. doi: 10.1073/pnas.0501074102 15774585 PMC555726

[39] Vasile F, Dossi E, Rouach N. Human astrocytes: structure and functions in the healthy brain. Brain Struct Funct. 2017;222(5):2017–29. doi: 10.1007/s00429-017-1383-5 28280934 PMC5504258

[40] Mayo L, Trauger SA, Blain M, Nadeau M, Patel B, Alvarez JI, et al. Regulation of astrocyte activation by glycolipids drives chronic CNS inflammation. Nat Med. 2014;20(10):1147–56. doi: 10.1038/nm.3681 25216636 PMC4255949

[41] Hidano S, Randall LM, Dawson L, Dietrich HK, Konradt C, Klover PJ, et al. STAT1 Signaling in Astrocytes Is Essential for Control of Infection in the Central Nervous System. mBio. 2016;7(6):e01881-16. doi: 10.1128/mBio.01881-16 27834206 PMC5101356

[42] He L, Zhang R, Yang M, Lu M. The role of astrocyte in neuroinflammation in traumatic brain injury. Biochim Biophys Acta Mol Basis Dis. 2024;1870(3):166992. doi: 10.1016/j.bbadis.2023.166992 38128844

[43] Amaducci L, Forno KI, Eng LF. Glial fibrillary acidic protein in cryogenic lesions of the rat brain. Neurosci Lett. 1981;21(1):27–32. doi: 10.1016/0304-3940(81)90052-5 7207867

[44] Iovino F, Orihuela CJ, Moorlag HE, Molema G, Bijlsma JJE. Interactions between blood-borne Streptococcus pneumoniae and the blood-brain barrier preceding meningitis. PLoS One. 2013;8(7):e68408. doi: 10.1371/journal.pone.0068408 23874613 PMC3713044

[45] Birla H, Xia J, Gao X, Zhao H, Wang F, Patel S, et al. Toll-like receptor 4 activation enhances Orai1-mediated calcium signal promoting cytokine production in spinal astrocytes. Cell Calcium. 2022;105:102619. doi: 10.1016/j.ceca.2022.102619 35780680 PMC9928533

[46] Li S, Fang Y, Zhang Y, Song M, Zhang X, Ding X, et al. Microglial NLRP3 inflammasome activates neurotoxic astrocytes in depression-like mice. Cell Rep. 2022;41(4):111532. doi: 10.1016/j.celrep.2022.111532 36288697

[47] Fang X, Wang H, Zhuo Z, Tian P, Chen Z, Wang Y, et al. miR-141-3p inhibits the activation of astrocytes and the release of inflammatory cytokines in bacterial meningitis through down-regulating HMGB1. Brain Res. 2021;1770:147611. doi: 10.1016/j.brainres.2021.147611 34403663

[48] Fischer S, Nasyrov E, Brosien M, Preissner KT, Marti HH, Kunze R. Self-extracellular RNA promotes pro-inflammatory response of astrocytes to exogenous and endogenous danger signals. J Neuroinflammation. 2021;18(1):252. doi: 10.1186/s12974-021-02286-w 34727934 PMC8561902

[49] Jin Y, Yao Y, El-Ashram S, Tian J, Shen J, Ji Y. The Neurotropic Parasite Toxoplasma gondii Induces Astrocyte Polarization Through NFκB Pathway. Front Med (Lausanne). 2019;6:267. doi: 10.3389/fmed.2019.00267 31803748 PMC6877604

[50] Hou B, Zhang Y, Liang P, He Y, Peng B, Liu W, et al. Inhibition of the NLRP3-inflammasome prevents cognitive deficits in experimental autoimmune encephalomyelitis mice via the alteration of astrocyte phenotype. Cell Death Dis. 2020;11(5):377. doi: 10.1038/s41419-020-2565-2 32415059 PMC7229224

[51] Huang W, Zheng X, Huang Q, Weng D, Yao S, Zhou C, et al. Protein Kinase CK2 Promotes Proliferation, Abnormal Differentiation, and Proinflammatory Cytokine Production of Keratinocytes via Regulation of STAT3 and Akt Pathways in Psoriasis. Am J Pathol. 2023;193(5):567–78. doi: 10.1016/j.ajpath.2023.01.016 37080661

[52] Trembley JH, Kren BT, Afzal M, Scaria GA, Klein MA, Ahmed K. Protein kinase CK2 - diverse roles in cancer cell biology and therapeutic promise. Mol Cell Biochem. 2023;478(4):899–926. doi: 10.1007/s11010-022-04558-2 36114992 PMC9483426

[53] Lian H, Su M, Zhu Y, Zhou Y, Soomro SH, Fu H. Protein Kinase CK2, a Potential Therapeutic Target in Carcinoma Management. Asian Pac J Cancer Prev. 2019;20(1):23–32. doi: 10.31557/APJCP.2019.20.1.23 30677865 PMC6485562

[54] Dörfel MJ, Westphal JK, Bellmann C, Krug SM, Cording J, Mittag S, et al. CK2-dependent phosphorylation of occludin regulates the interaction with ZO-proteins and tight junction integrity. Cell Commun Signal. 2013;11(1):40. doi: 10.1186/1478-811X-11-40 23758859 PMC3695765

[55] Ceyzériat K, Abjean L, Carrillo-de Sauvage M-A, Ben Haim L, Escartin C. The complex STATes of astrocyte reactivity: How are they controlled by the JAK-STAT3 pathway? Neuroscience. 2016;330:205–18. doi: 10.1016/j.neuroscience.2016.05.043 27241943

[56] Brambilla R, Bracchi-Ricard V, Hu W-H, Frydel B, Bramwell A, Karmally S, et al. Inhibition of astroglial nuclear factor kappaB reduces inflammation and improves functional recovery after spinal cord injury. J Exp Med. 2005;202(1):145–56. doi: 10.1084/jem.20041918 15998793 PMC2212896

[57] Roy Choudhury G, Ryou M-G, Poteet E, Wen Y, He R, Sun F, et al. Involvement of p38 MAPK in reactive astrogliosis induced by ischemic stroke. Brain Res. 2014;1551:45–58. doi: 10.1016/j.brainres.2014.01.013 24440774 PMC3987968

[58] Furman JL, Norris CM. Calcineurin and glial signaling: neuroinflammation and beyond. J Neuroinflammation. 2014;11:158. doi: 10.1186/s12974-014-0158-7 25199950 PMC4172899

[59] Quotti Tubi L, Canovas Nunes S, Brancalion A, Doriguzzi Breatta E, Manni S, Mandato E, et al. Protein kinase CK2 regulates AKT, NF-κB and STAT3 activation, stem cell viability and proliferation in acute myeloid leukemia. Leukemia. 2017;31(2):292–300. doi: 10.1038/leu.2016.209 27479180

[60] Pecci A, Ma X, Savoia A, Adelstein RS. MYH9: Structure, functions and role of non-muscle myosin IIA in human disease. Gene. 2018;664:152–67. doi: 10.1016/j.gene.2018.04.048 29679756 PMC5970098

[61] Even-Ram S, Doyle AD, Conti MA, Matsumoto K, Adelstein RS, Yamada KM. Myosin IIA regulates cell motility and actomyosin-microtubule crosstalk. Nat Cell Biol. 2007;9(3):299–309. doi: 10.1038/ncb1540 17310241

[62] Kohlstedt K, Kellner R, Busse R, Fleming I. Signaling via the angiotensin-converting enzyme results in the phosphorylation of the nonmuscle myosin heavy chain IIA. Mol Pharmacol. 2006;69(1):19–26. doi: 10.1124/mol.105.016733 16186248

[63] Bonnet H, Filhol O, Truchet I, Brethenou P, Cochet C, Amalric F, et al. Fibroblast growth factor-2 binds to the regulatory beta subunit of CK2 and directly stimulates CK2 activity toward nucleolin. J Biol Chem. 1996;271(40):24781–7. doi: 10.1074/jbc.271.40.24781 8798749

[64] Yang B, Wu A, Hu Y, Tao C, Wang JM, Lu Y, et al. Mucin 17 inhibits the progression of human gastric cancer by limiting inflammatory responses through a MYH9-p53-RhoA regulatory feedback loop. J Exp Clin Cancer Res. 2019;38(1):283. doi: 10.1186/s13046-019-1279-8 31262330 PMC6604468

