# Supplementary figures and images for: CK2 derived from brain microvascular endothelial cells induces astrocyte inflammatory response in Escherichia coli-induced meningitis
Source: PLoS Pathog. 2025 Sep 10;21(9):e1013464. doi: 10.1371/journal.ppat.1013464 (PMC12422478; doi:10.1371/journal.ppat.1013464)

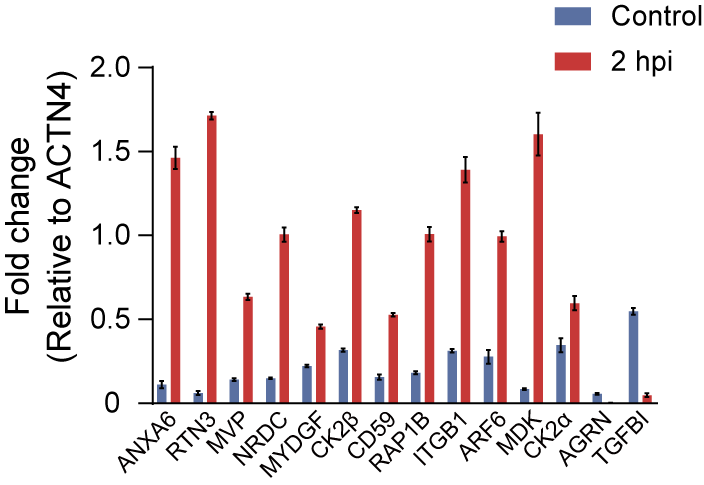

Supplement: S2 Fig — Analysis results are presented as DEPs/loading control (ACTN4). n = 3. Data are shown as mean ± SEM. (TIF) [file ppat.1013464.s002.tif]

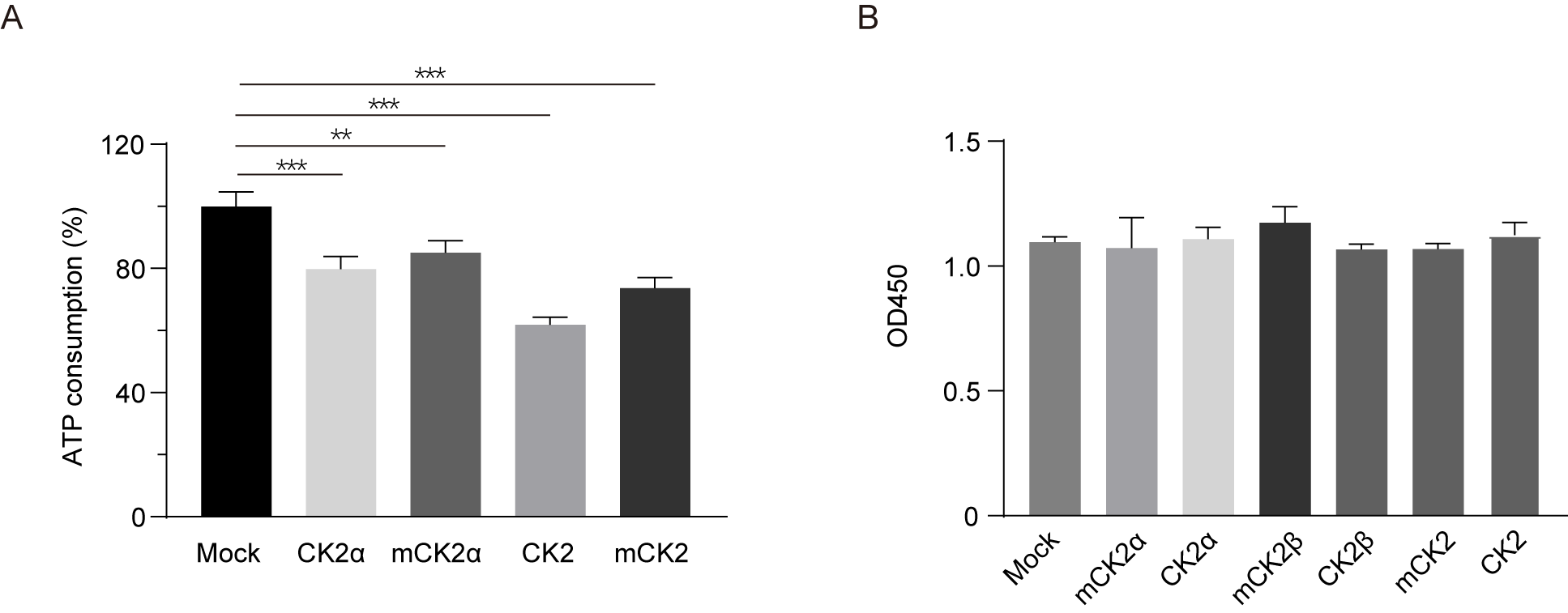

Supplement: S3 Fig — (A) Enzyme activity of CK2 subunits and holoenzyme was reflected by ATP consumption after the incubation of 50 ng CK2α, mCK2α, CK2 and mCK2 with equivalent U251 cell lysates for 10 min at 37 °C. n = 3. Data are shown as mean ± SEM. One-way ANOVA followed by Tukey’s multiple comparison. **p < 0.002, ***p < 0.001. (B) Cytotoxicity of CK2 subunits and holoenzyme was reflected by cell viability measured with a Cell Counting Kit-8. Astrocytes in 96-well plates were treated by 400 ng/mL mCK2α, CK2α, mCK2β, CK2β, mCK2 and CK2 for 1 h, five parallel wells were used for each treatment. (TIF) [file ppat.1013464.s003.tif]

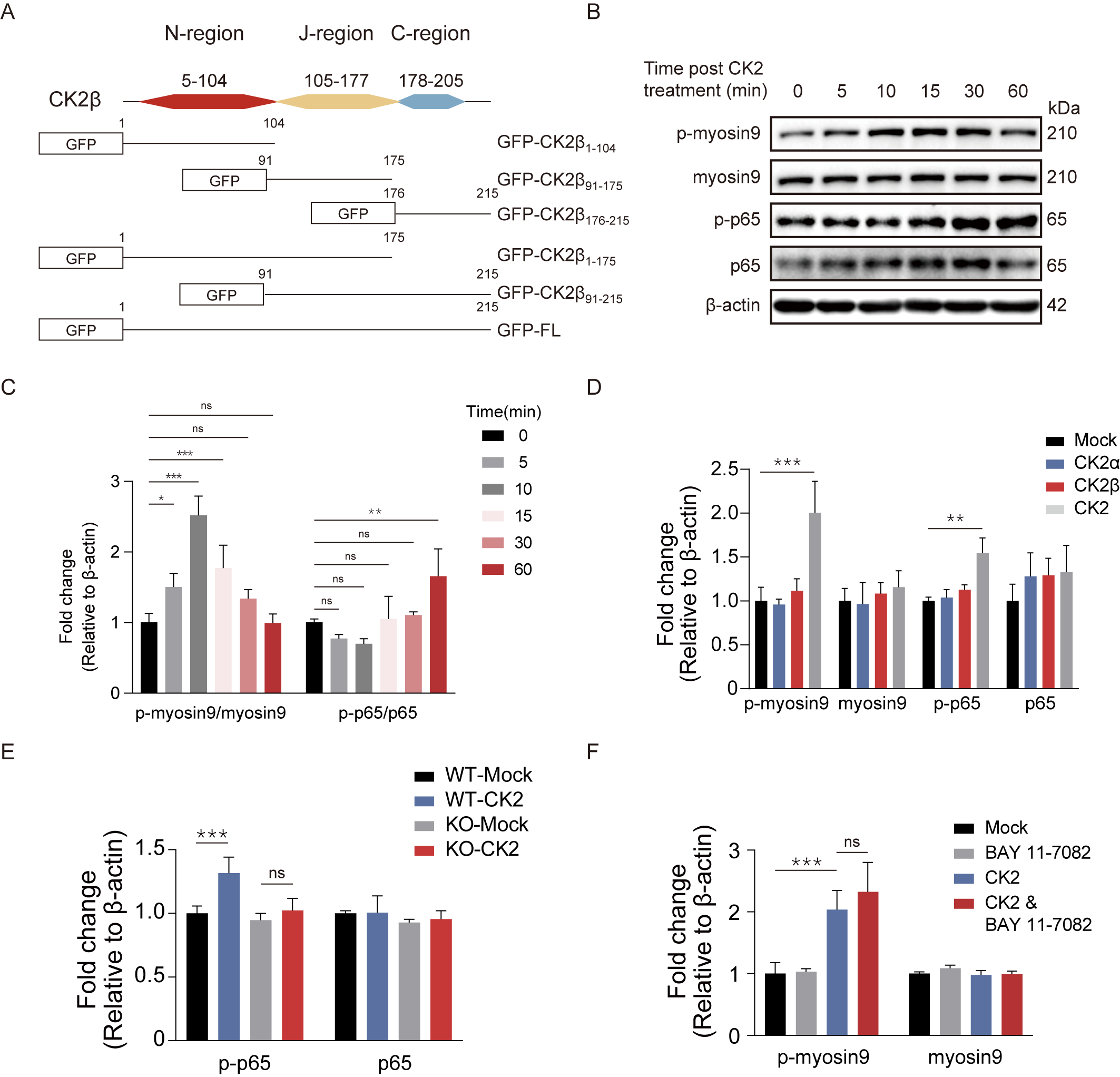

Supplement: S4 Fig — (A) Illustration of CK2β truncated mutant constructs with amino-terminal GFP fusions. (B) Western blot of astrocytic p65/myosin 9 phosphorylation induced by CK2 in a time gradient in mouse primary astrocytes. n = 3. (C) Fold changes of phosphorylated myosin 9/myosin 9 and phosphorylated p65/p65 in mouse primary astrocytes after CK2 treatment. n = 3. (D) Fold changes of phosphorylated myosin 9, myosin 9, phosphorylated p65 and p65 in mouse primary astrocytes at 30 min after 100 ng/mL CK2α, CK2β and CK2 treatment. n = 3. (E) Fold changes of myosin 9, phosphorylated p65 and p65 in WT and myosin 9-KO U251 cells after 100 ng/mL CK2 treatment for 1 h. n = 3. (F) Fold changes of phosphorylated myosin 9 and myosin 9 in mouse primary astrocytes pretreated with BAY 11–7082 (NF-κB inhibitor) or vehicle (DMSO) following 100 ng/mL CK2 treatment for 10 min. n = 3. Data are shown as mean ± SEM. Two-way ANOVA (C, D, E, F) followed by Bonferroni’s multiple comparison tests; *p < 0.033, ** p < 0.002, *** p < 0.001, ns, not significant. (TIF) [file ppat.1013464.s004.tif]

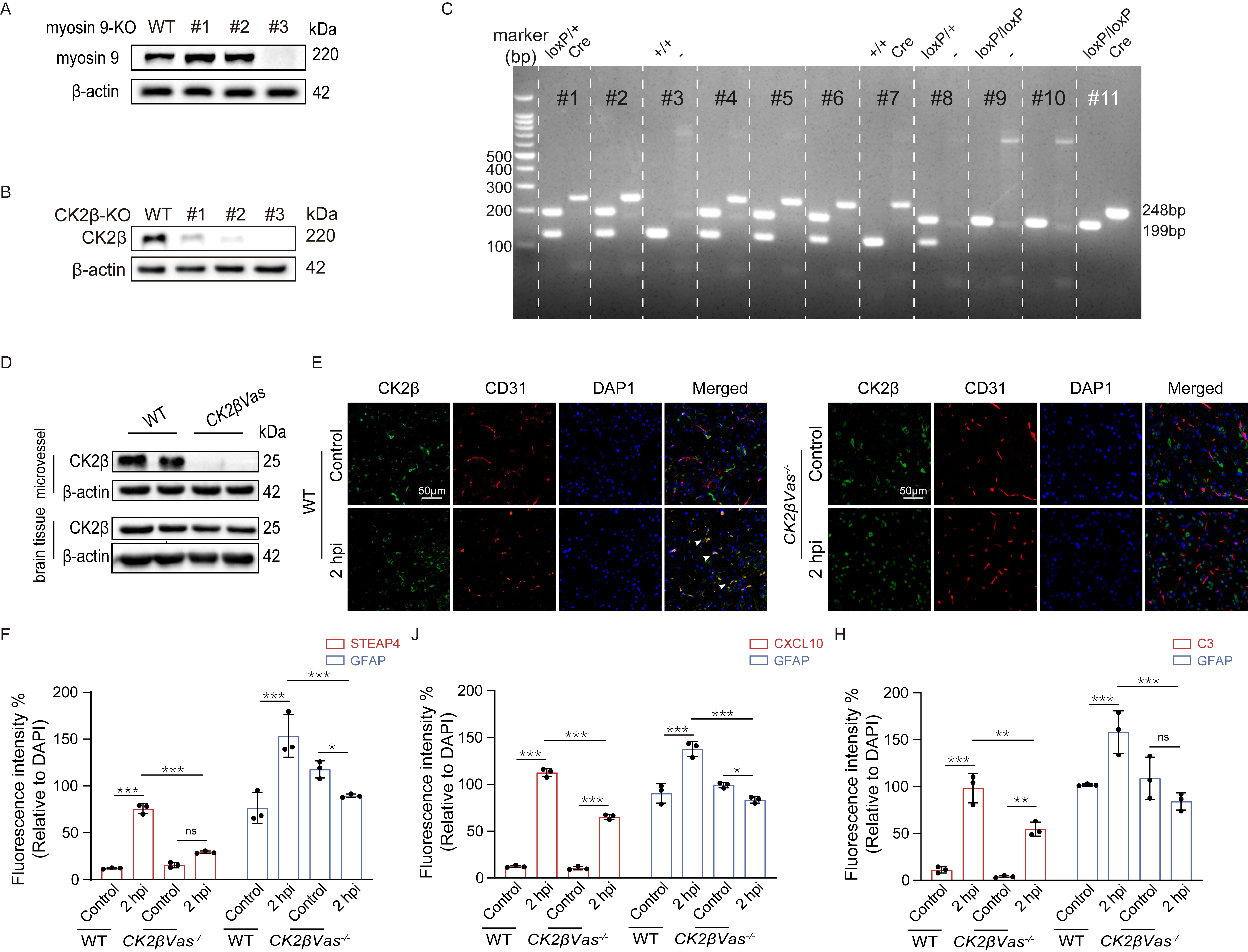

Supplement: S5 Fig — (A)Western blot results of myosin 9-KO U251 cell line screening. n = 3. (B) Western blot results of CK2β-KO hBMEC cell line screening. n = 3. (C) PCR-based genotyping screening for CK2β conditional knockout mice, which detected the DNA bands at 199 bp for homozygotes with loxP sites in CSNK2B gene and 248 bp for Cre recombinase (for example, #11). (D) CK2β expression in cerebral microvessel samples and brain tissue samples from WT and CK2βVas-/- mice. n = 3. (E) Immunostaining of CK2β (green) and the vascular endothelial marker CD31 (red) in the brain parenchyma of WT and CK2βVas-/-mice 2 h after infection. Scale bar, 50 μm. Quantification of (F) STEAP4, (G) CXCL10, and (H) C3 (all in red) with GFAP (green) in the hippocampus of WT and CK2βVas-/- mice post 2 h infection. Scale bar, 20 μm. n = 3. Data are shown as mean ± SEM. Two-way ANOVA (F, G, H) followed by Bonferroni’s multiple comparison test. *p < 0.033, ** p < 0.002, *** p < 0.001, ns, not significant. (TIF) [file ppat.1013464.s005.tif]

Figure 1

A

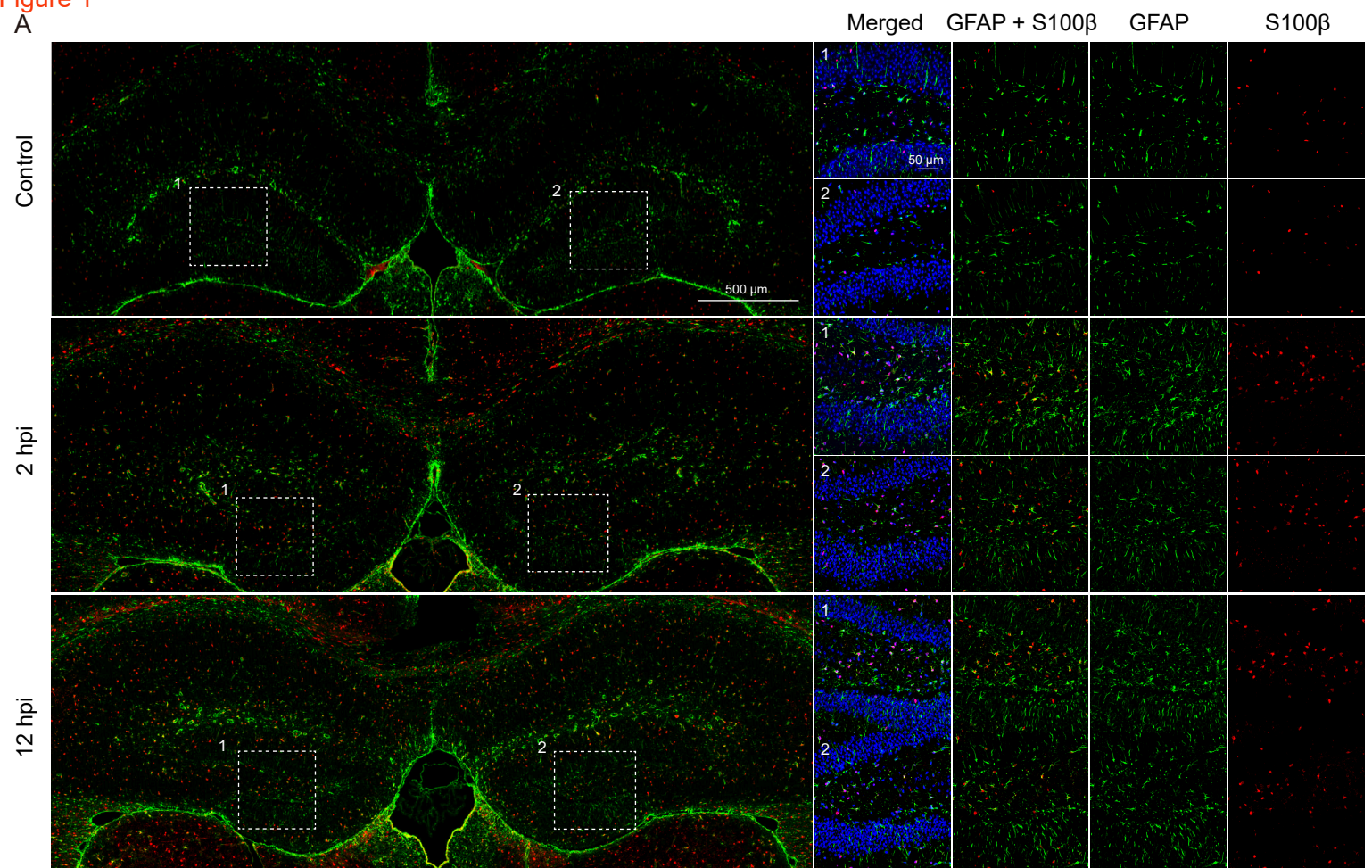

D

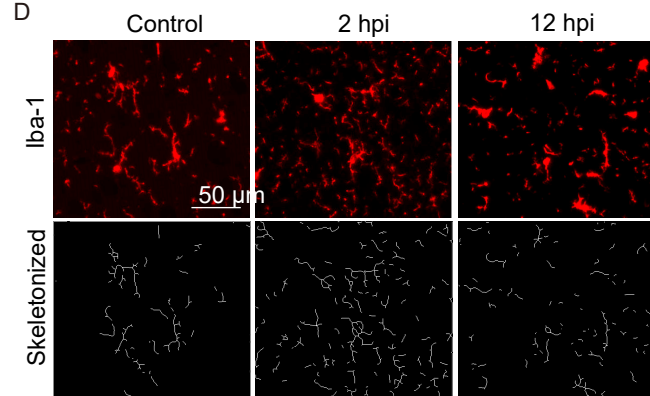

Figure 3

E

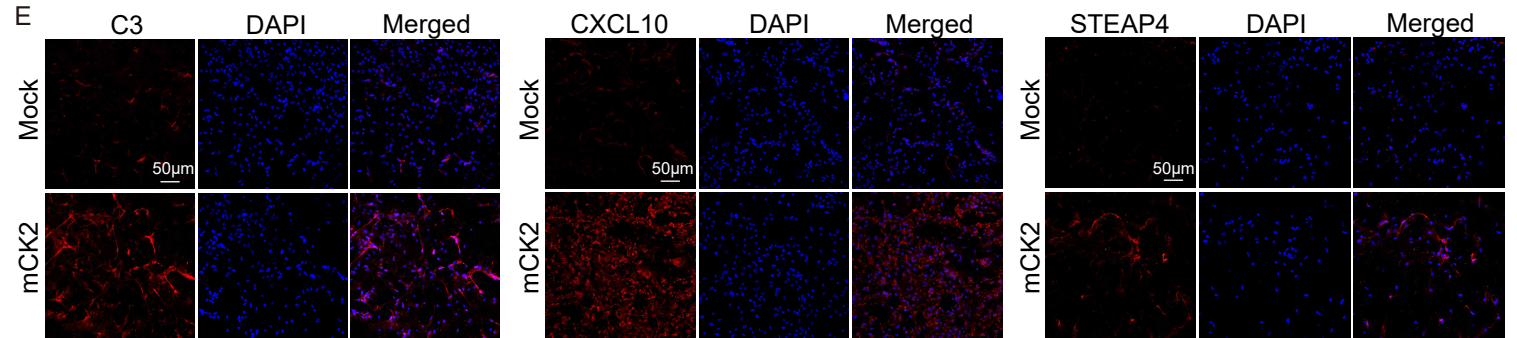

Figure 5

C

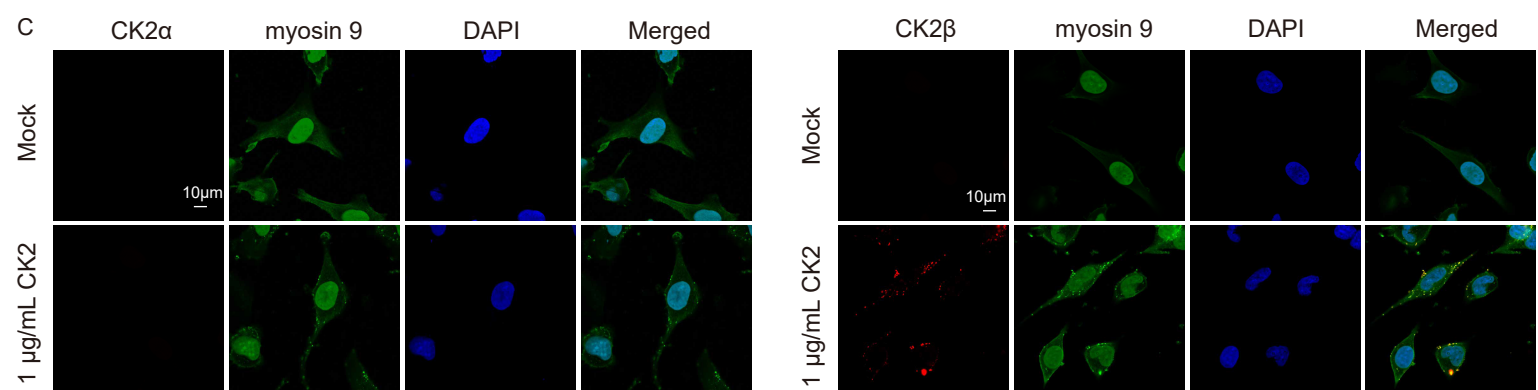

Figure 6

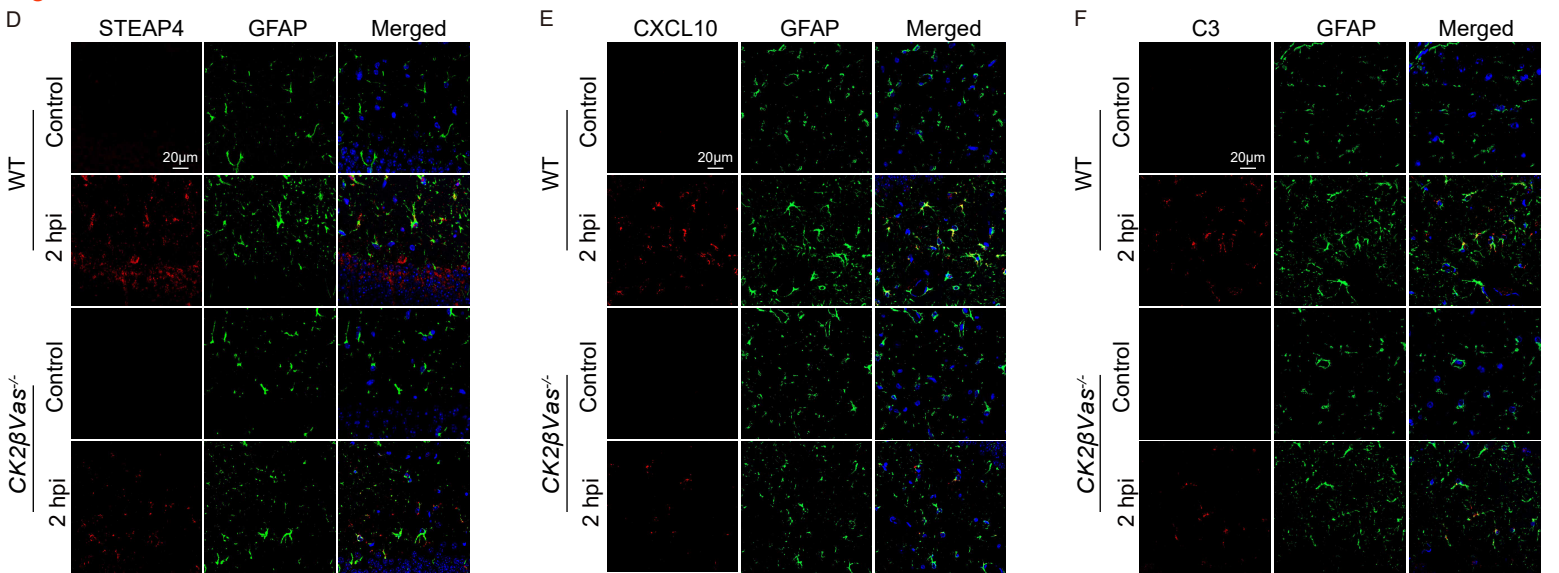

Supplement: S2 Data — (PDF) [file ppat.1013464.s012.pdf]
