# Supplementary material for: CK2 derived from brain microvascular endothelial cells induces astrocyte inflammatory response in Escherichia coli-induced meningitis
Source: PLoS Pathog. 2025 Sep 10;21(9):e1013464. doi: 10.1371/journal.ppat.1013464 (PMC12422478; doi:10.1371/journal.ppat.1013464)
Supplement: S1 Table — (DOCX) [file ppat.1013464.s006.docx]

**CK2 derived from brain microvascular endothelial cells induces astrocyte inflammatory response in *Escherichia coli*-induced meningitis**

**S1 Table. Primers, sgRNA and siRNA used in the study.**

| Primer | Forward sequence (5’-3’) | Reverse sequence (5’-3’) |
| --- | --- | --- |
| hTNF-α | CCTCTTCTCCTTCCTGATC | TTGCTACAACATGGGCTA |
| hIL-1β | AGGATATGGAGCAACAAGT | GCAGGACAGGTACAGATT |
| hIL-8 | CATACTCCAAACCTTTCCAC | AAACTTCTCCACAACCCT |
| hMIP2 | AGTGTGAAGGTGAAGTCC | CTTTCTGCCCATTCTTGAG |
| hCCL2 | CAGCCAGATGCAATCAATGCC | TGGAATCCTGAACCCACTTCT |
| hCXCL3 | CGCCCAAACCGAAGTCATAG | GCTCCCCTTGTTCAGTATCTTTT |
| hCXCL10 | GTGGCATTCAAGGAGTACCTC | TGATGGCCTTCGATTCTGGATT |
| hGAPDH | CAACAGCCTCAAGATCATCAG | GAGTCCTTCCACGATACCA |
| mTNFα | CCCTCACACTCAGATCATCTTCT | GCTACGACGTGGGCTACAG |
| mIL-1β | GCAACTGTTCCTGAACTCAACT | ATCTTTTGGGGTCCGTCAACT |
| mIL-6 | TAGTCCTTCCTACCCCAATTTCC | TTGGTCCTTAGCCACTCCTTC |
| mLCN2 | CCACCTCAGACCTGATCCCA | CCCCTGGAATTGGTTGTCCTG |
| mSTEAP4 | CCGTTACCCAGGCAATACTC | TCTCCAGTTGACTGCATTGC |
| Primer | Forward sequence (5’-3’) | Reverse sequence (5’-3’) |
| mS1PR3 | GGATGTGCTGGCTCATTGC | CAGGATGGTAGAGCAGTCAGG |
| mOSMR | AATGTCAGTGAAGGCATGAAAGG | GAAGGTTGTTTAGACCACCCC |
| mASPG | AGGCATCCGGGTGTCATTG | TTCGAGGTCGGCCCTGTAT |
| mGFAP | CTGCGGCTCGATCAACTCA | TCCAGCGACTCAATCTTCCTC |
| mCXCL10 | CCCACGTGTTGAGATCATTG | CACTGGGTAAAGGGGAGTGA |
| mH2-T23 | GGACCGCGAATGACATAGC | GCACCTCAGGGTGACTTCAT |
| mH2-D1 | TCCGAGATTGTAAAGCGTGAAGA | ACAGGGCAGTGCAGGGATAG |
| mSrgn | GCAAGGTTATCCTGCTCGGA | TGGGAGGGCCGATGTTATTG |
| mPsmb8 | CAGTCCTGAAGAGGCCTACG | CACTTTCACCCAACCGTCTT |
| mAmigo2 | GAGGCGACCATAATGTCGTT | GCATCCAACAGTCCGATTCT |
| mC3 | CCAGCTCCCCATTAGCTCTG | GCACTTGCCTCTTTAGGAAGTC |
| mIigp1 | GGGGCAATAGCTCATTGGTA | ACCTCGAAGACATCCCCTTT |
| mActb | GGCTGTATTCCCCTCCATCG | CCAGTTGGTAACAATGCCATGT |
| His-hCK2α | GGAATTCCATATGATGTCGGGACCCGTGC | CCCAAGCTTCTATTACTGCTGAGCGCCAG |
| GST-hCK2β | CGGGATCCATGAGCAGCTCAGAGGAGGT | CCGCTCGAGTCAGCGAATCGTCTTGACTG |
| Primer | Forward sequence (5’-3’) | Reverse sequence (5’-3’) |
| His-mCK2α | GGAATTCCATATGTCGGGACCCGTGCC | CGGGATCCTTACTGCTGAGCGCCAGCA |
| GST-mCK2β | CGGGATCCATGAGCAGCTCAGAGGAGGT | CCGCTCGAGTCAGCGAATCGTCTTGACTG |
| GST-hCK2β | CGGGATCCATGAGCAGCTCAGAGGAGGT | CCGCTCGAGTCAGCGAATCGTCTTGACTG |
| GFP-CK2β_1-104_ | CCGCTCGAGCAATGAGCAGCTCAGAGGAG | CCCAAGCTTTCCTTGCTGGTACTTTTCC |
| GFP-CK2β_91-175_ | CCGCTCGAGCAAACCGTGGCATCGC | CCCAAGCTTCCGGTACTCGGGATGC |
| GFP-CK2β_176-215_ | CCGCTCGAGCACCCAAGAGACCTGCCA | CCCAAGCTTTCAGCGAATCGTCTTGACT |
| GST-MYH9_1897-1935_ | CCGCTCGAGCTCTGGAAGTTCTGTTCCAGGGGCCTCAGCGCGAGCTGGAGGAC | CGCGGATCCAGCGTAATCTGGAACATCGTATGGGTAGGCCATTCGGCGGGGC |
| CK2βsgRNA-1 | AGTAGGCGCCATCCGTGTGA | TCACACGGATGGCGCCTACT |
| CK2βsgRNA-2 | CGCTACATCCTTACCAACCG | CGGTTGGTAAGGATGTAGCG |
| Myosin9 sgRNA-1 | ACGCCACGTACGCCAGATAC | GTATCTGGCGTACGTGGCGT |
| Myosin9 sgRNA-2 | CACGTGCCTCAACGAAGCCT | AGGCTTCGTTGAGGCACGTG |
