# Supplementary material for: CK2 derived from brain microvascular endothelial cells induces astrocyte inflammatory response in Escherichia coli-induced meningitis
Source: PLoS Pathog. 2025 Sep 10;21(9):e1013464. doi: 10.1371/journal.ppat.1013464 (PMC12422478; doi:10.1371/journal.ppat.1013464)
Supplement: S5 Table — (DOCX) [file ppat.1013464.s010.docx]

**CK2 derived from brain microvascular endothelial cells induces astrocyte inflammatory response in *Escherichia coli*-induced meningitis**

**S5 Table. Antibodies used in the study.**

| Antibodies | Source | Cat No. / RRID | Dilution rate |
| --- | --- | --- | --- |
| Mouse monoclonal anti-IBA1 | Proteintech | 66827-1-Ig; RRID: AB_2882170 | 1:1000 (IHC) |
| Rabbit polyclonal anti-S100β | Proteintech | 15146-1-AP; RRID: AB_2254244 | 1:1000 (IHC) |
| Mouse monoclonal anti-GFAP | Proteintech | 60190-1-Ig; RRID: AB_10838694 | 1:500 (IHC); 1:5000 (Western blot) |
| Rabbit polyclonal anti-CD31 | Proteintech | 11265-1-AP; RRID: AB_2299349 | 1:2000 (IHC) |
| Rabbit polyclonal anti-Albumin | Proteintech | 16475-1-AP; RRID: AB_2242567 | 1:1000 (IHC) |
| Rabbit polyclonal anti-ANXA6 | Proteintech | 12542-1-AP; RRID: AB_2057753 | 1:1000 (Western blot) |
| Rabbit polyclonal anti-RTN3 | Proteintech | 12055-2-AP; RRID: AB_2301357 | 1:1000 (Western blot) |
| Rabbit polyclonal anti-MVP | Proteintech | 16478-1-AP; RRID: AB_2147597 | 1:500 (Western blot) |
| Rabbit polyclonal anti-NRDC | Proteintech | 15630-1-AP; RRID: AB_2154525 | 1:500 (Western blot) |
| Rabbit polyclonal anti-MYDGF | Proteintech | 11353-1-AP; RRID: AB_2207647 | 1:500 (Western blot) |
| Rabbit polyclonal anti-CK2α | Abcepta | AP8144C; RRID: AB_2087332 | 1:500 (Western blot) |
| Rabbit polyclonal anti-CK2β | Proteintech | 20234-1-AP; RRID: AB_10733246 | 1:1000 (Western blot); 1:200 (IF) |
| Rabbit polyclonal anti-CD59 | Abcepta | AP22266c; RRID: AB_3075422 | 1:500 (Western blot) |
| Rabbit polyclonal anti-RAP1B | Proteintech | 10840-1-AP; RRID: AB_2253539 | 1:1000 (Western blot) |
| Rabbit polyclonal anti-ITGB1 | Proteintech | 12594-1-AP; RRID: AB_2130085 | 1:5000 (Western blot) |
| Rabbit polyclonal anti-ARF6 | Proteintech | 20225-1-AP; RRID: AB_10646481 | 1:1000 (Western blot) |
| Rabbit polyclonal anti-MDK | Abcepta | AP12076b; RRID: AB_10818731 | 1:1000 (Western blot) |
| Rabbit polyclonal anti-AGRN | Abclonal | A17320; RRID: AB_2768283 | 1:1000 (Western blot) |
| Rabbit monoclonal anti-TGFBI | Abclonal | A2407; RRID: AB_2863002 | 1:1000 (Western blot) |
| Rabbit polyclonal anti-ACTN4 | Proteintech | 19096-1-AP; RRID: AB_10642150 | 1:5000 (Western blot) |
| Rabbit monoclonal anti-Phospho-p65 | Cell Signaling Technology | 3033S; RRID: AB_331284 | 1:1000 (Western blot) |
| Rabbit monoclonal anti-p65 | Cell Signaling Technology | 6956S; RRID: AB_10828935 | 1:1000 (Western blot) |
| Mouse monoclonal anti-β-actin | Proteintech | 66009-1-Ig; RRID: AB_2687938 | 1:5000 (Western blot) |
| Rabbit monoclonal anti-phospho-myosin9 | Abmart | TA4425S; RRID: AB_3075418 | 1:1000 (Western blot) |
| Rabbit polyclonal anti-myosin9 | Proteintech | 11128-1-AP; RRID: AB_2147294 | 2 μg for 500 μg total protein lysate (IP); 1:5000 (Western blot); 1:200 (IF) |
| Rabbit polyclonal anti-CXCL10 | Proteintech | 10937-1-AP; RRID: AB_2088002 | 1:200 (IHC); 1:200 (IF) |
| Rabbit polyclonal anti-C3 | Proteintech | 21337-1-AP; RRID: AB_2878843 | 1:1000 (IHC); 1:200 (IF) |
| Rabbit polyclonal anti-STEAP4 | Abclonal | A17767; RRID: AB_2772430 | 1:100 (IHC); 1:100 (IF) |
| Mouse monoclonal anti-His tag | Proteintech | 66005-1-Ig; RRID: AB_11232599 | 0.5 μg for 500 μg total protein lysate (IP); 1:5000 (Western blot); 1:200 (IF) |
| Mouse monoclonal anti-GST tag | Proteintech | 66001-2-Ig; RRID: AB_2881488 | 0.5 μg for 500 μg total protein lysate (IP); 1:5000 (Western blot); 1:200 (IF) |
| Mouse monoclonal anti-GFP tag | Proteintech | 66002-1-Ig; RRID: AB_11182611 | 0.5 μg for 500 μg total protein lysate (IP); 1:2000 (Western blot) |
| HRP-conjugated Goat Anti-Rabbit IgG(H+L) | Biogragon | BF03008; RRID: AB_3073988 | 1:5000 (Western blot) |
| HRP-conjugated Goat Anti-Mouse IgG(H+L) | Biogragon | BF03001; N/A | 1:5000 (Western blot) |
| CoraLite488- conjugated Goat Anti-Mouse IgG(H+L) | Proteintech | SA00013-1; RRID: AB_2810983 | 1:200 (IHC); 1:200 (IF) |
| CoraLite488- conjugated Goat Anti- Rabbit IgG(H+L) | Proteintech | SA00013–2; RRID: AB_2797132 | 1:100 (IHC); 1:100 (IF) |
| Cy3- conjugated Affinipure Goat Anti-Mouse IgG(H+L) | Proteintech | SA00009-1; RRID: AB_2814746 | 1:50 (IHC); 1:50 (IF) |
| Cy3- conjugated Affinipure Goat Anti- Rabbit IgG(H+L) | Proteintech | SA00009-2; RRID: AB_2890957 | 1:50 (IHC); 1:50 (IF) |
