# Supplementary material for: CK2 derived from brain microvascular endothelial cells induces astrocyte inflammatory response in Escherichia coli-induced meningitis
Source: PLoS Pathog. 2025 Sep 10;21(9):e1013464. doi: 10.1371/journal.ppat.1013464 (PMC12422478; doi:10.1371/journal.ppat.1013464)

**CK2 derived from brain microvascular endothelial cells induces astrocyte inflammatory response in *Escherichia coli*-induced meningitis**

**S1 Data. Raw data of SDS-PAGE and western blot in this study.**


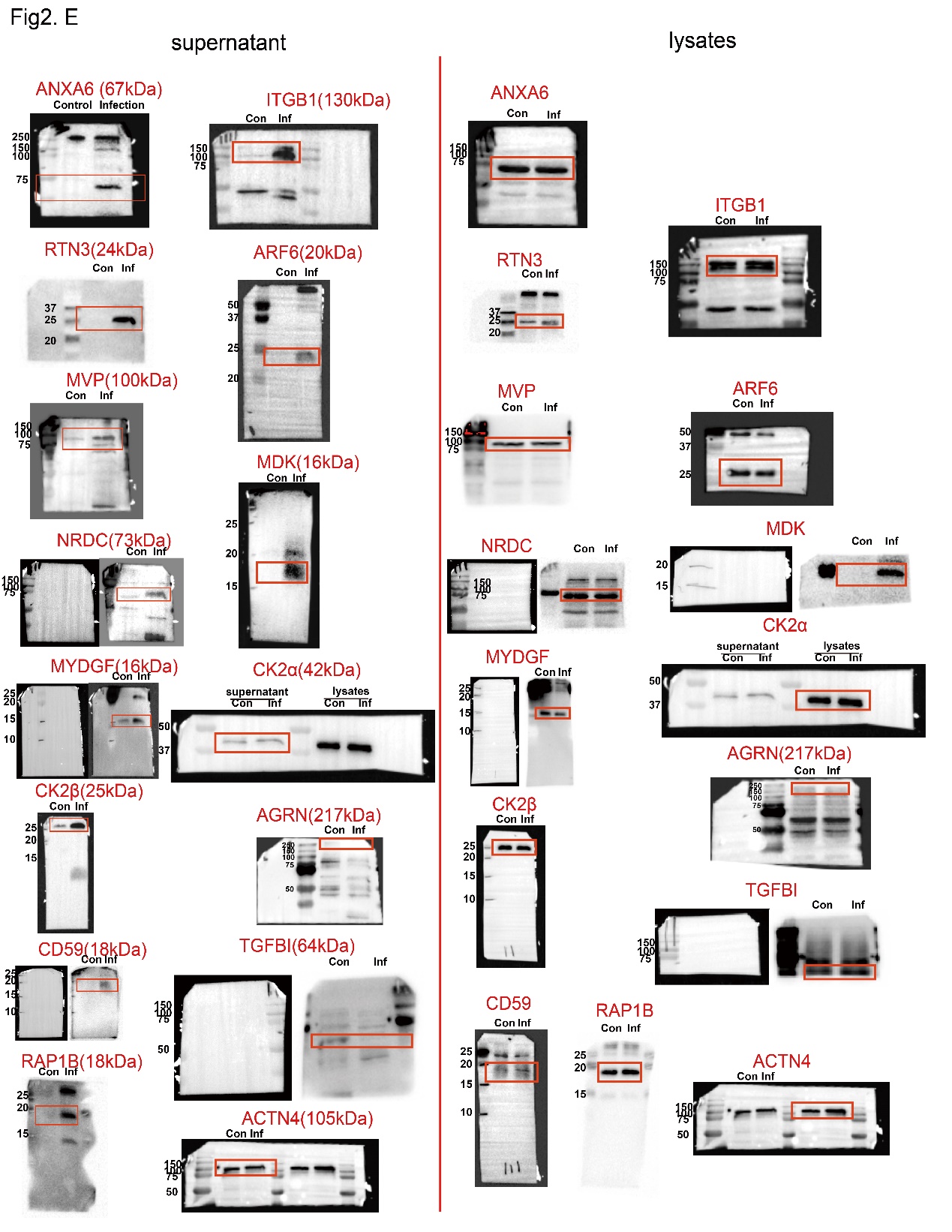

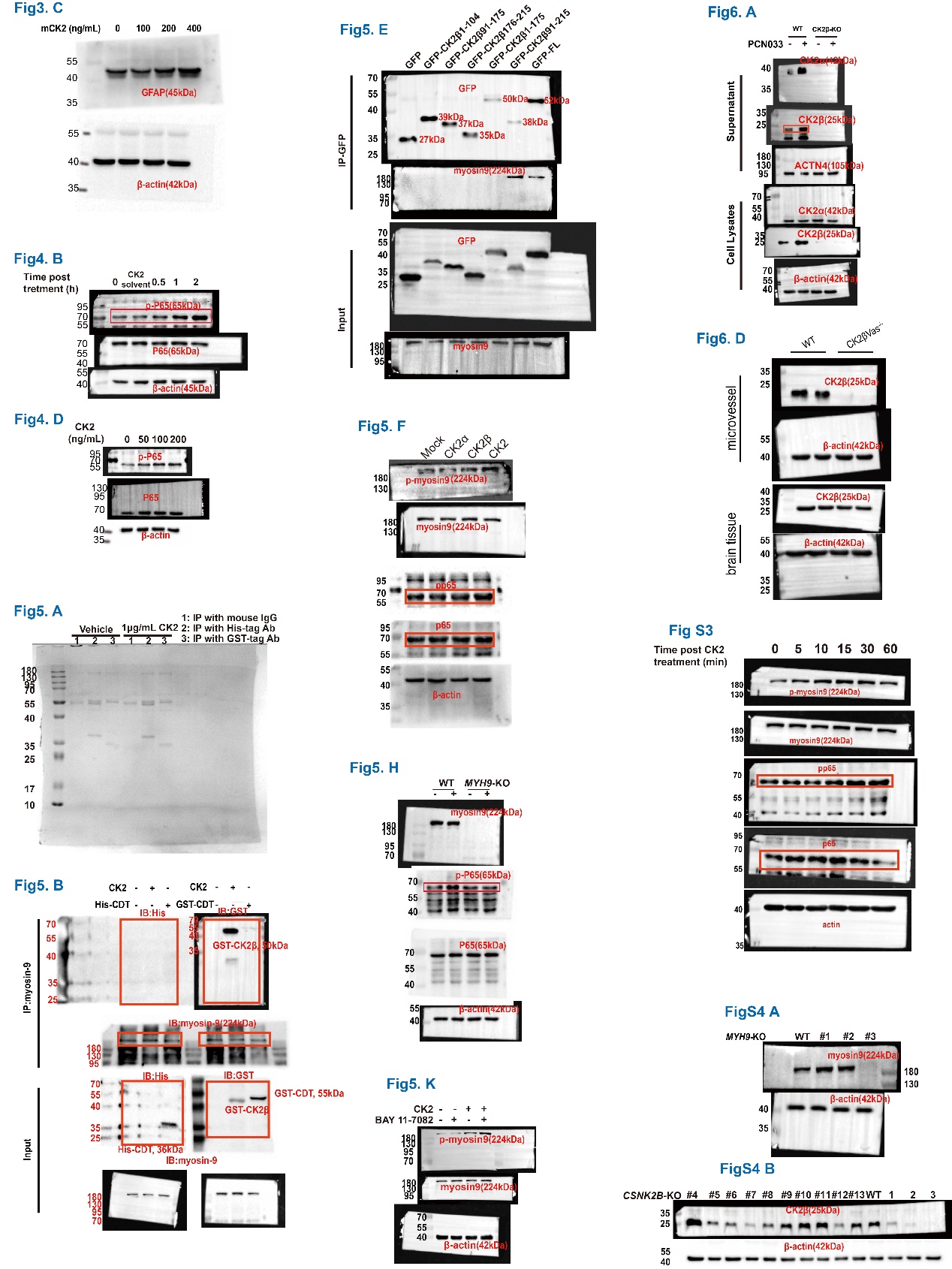

Supplement: S1 Data — (DOCX) [file ppat.1013464.s011.docx]
